# Supplementary material for: Magnetically Guided Macrophage Immunobots Coordinate Iron‐Metabolic Cascades and Immunogenic Ferroptosis for Tumor Immunotherapy
Source: Adv Sci (Weinh). 2026 Sep 27:e77760. Online ahead of print. doi: 10.1002/advs.77760 (PMC13616292; doi:10.1002/advs.77760)
Supplement: Supplementary file 1 [file ADVS-9999-e77760-s007.pdf]

Supplementary Materials for

Magnetically guided macrophage immunobots coordinate iron-metabolic cascades and immunogenic ferroptosis for tumor immunotherapy

*Xirui Zeng[1]†, Yingfei Zhang[2]†, Chong Wu[3], Qijia Duan[1], Mengqi Fan[2], Kuan Wang[2], Shirong Cai[3] and Immihan Ceren Yasa[1,2] **

This PDF file includes:

Text S1 to S2
Figs. S1 to S29
Tables S1 and S2
Legends for movies S1 to S7

Other Supplementary Material for this manuscript includes the following:

Movies S1 to S7

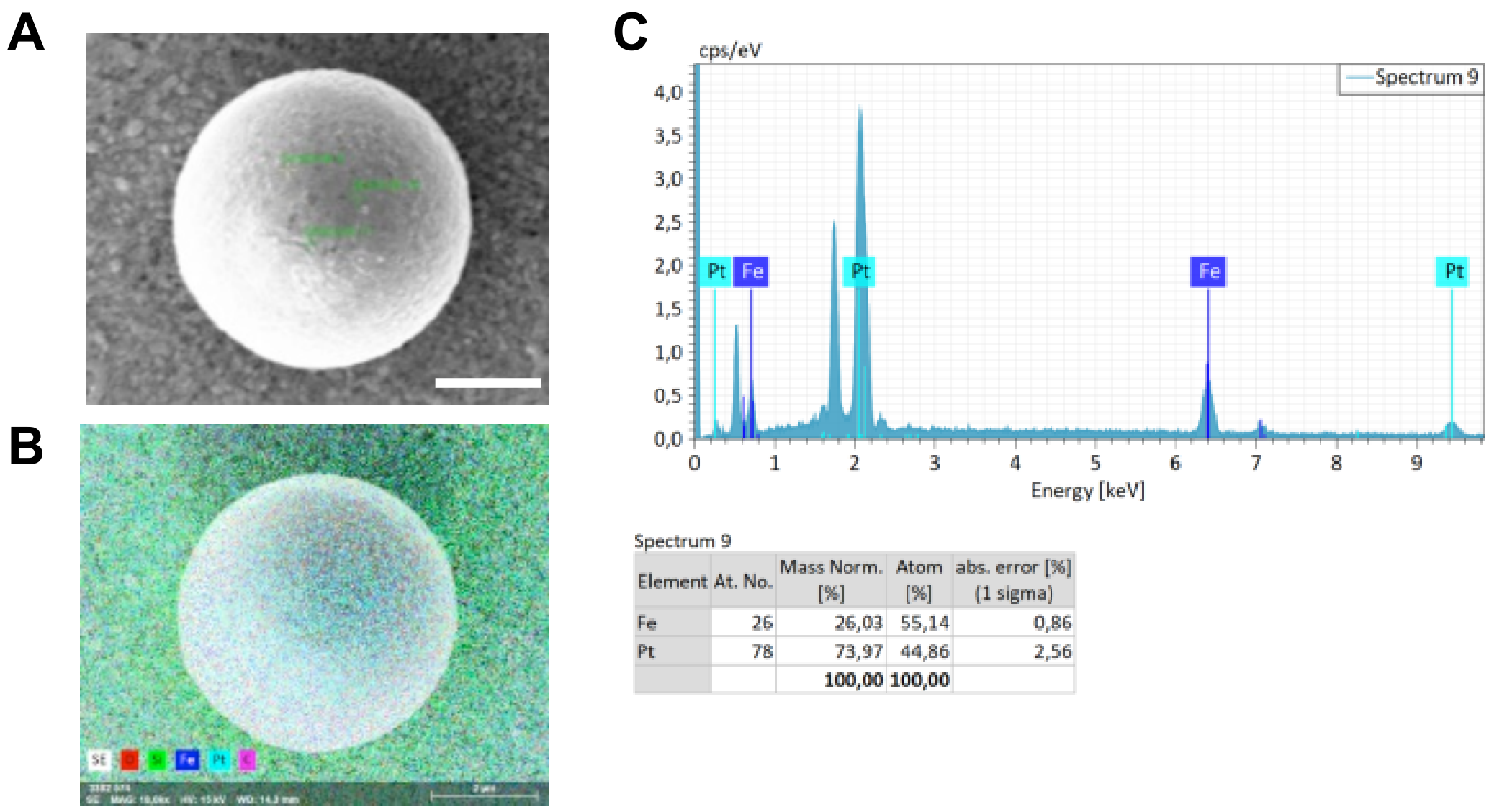

Element	At. No.	Mass Norm. [%]	Atom [%]	abs. error [%] (1 sigma)
Fe	26	26,03	55,14	0,86
Pt	78	73,97	44,86	2,56
		100,00	100,00	

Fig. S1. Morphological and elemental characterization of FePt-coated microrollers. (A) Representative SEM image with EDS point scanning showing the positions, (B) element mapping of a single microroller showing the Fe and Pt localization on the surface. (C) EDS spectra showing the Fe/Pt mass percentage and atomic ratio of each micro-zone. Scale bar, 2 µm.

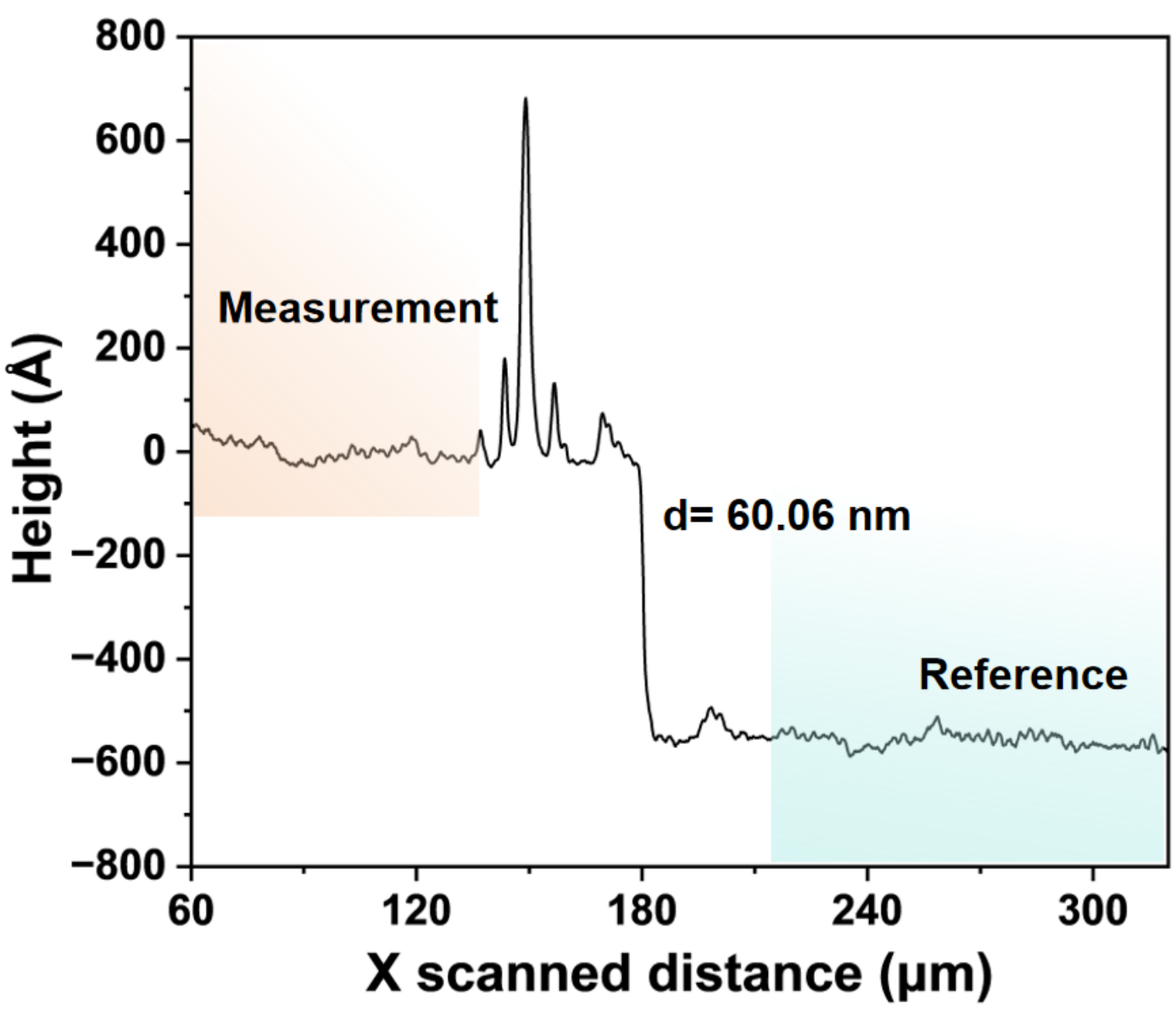

Fig. S2. Step profilometry measurement showing that the Fe200-Pt140 film thickness is 60.06 nm.

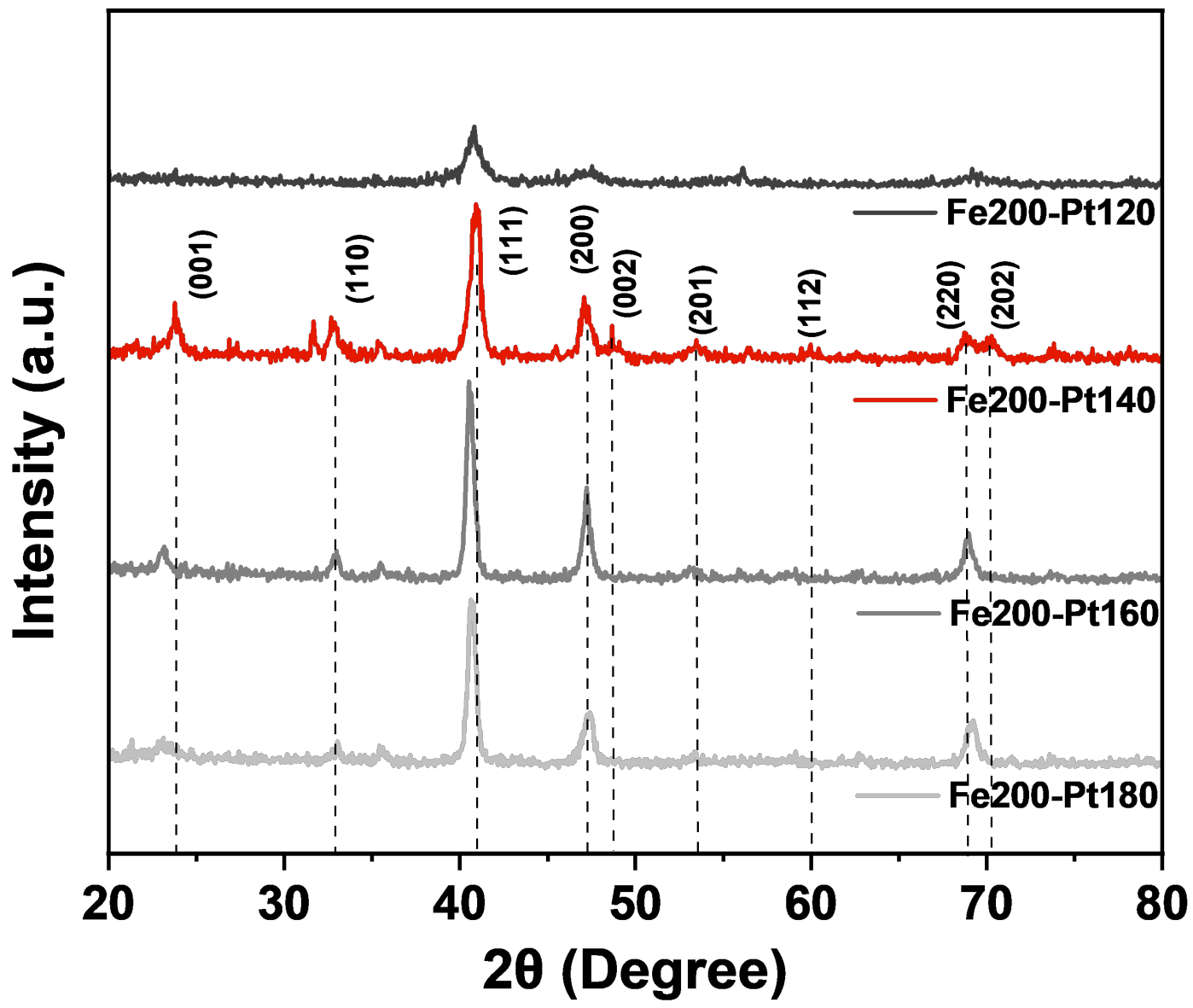

Fig. S3. XRD patterns of FePt films prepared under different Fe/Pt power ratios after annealing. Representative L10-FePt reflections are indicated in the Fe200-Pt140 group.

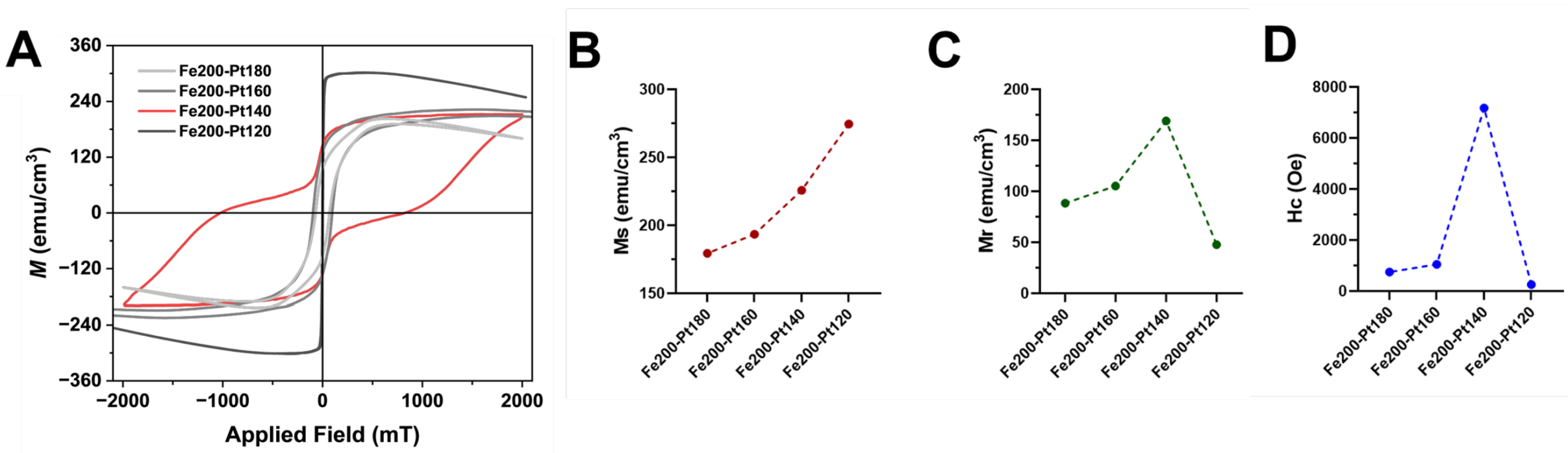

Fig. S4. Characterization of magnetic properties of the coated FePt using VSM. Hysteresis loops for different sputtering power groups. (A) Merged hysteresis loops. Changes in (B) Ms (saturation magnetization), (C) Mr (remanence) and (D) Hc (coercivity) among different groups after altering the FePt power ratio.

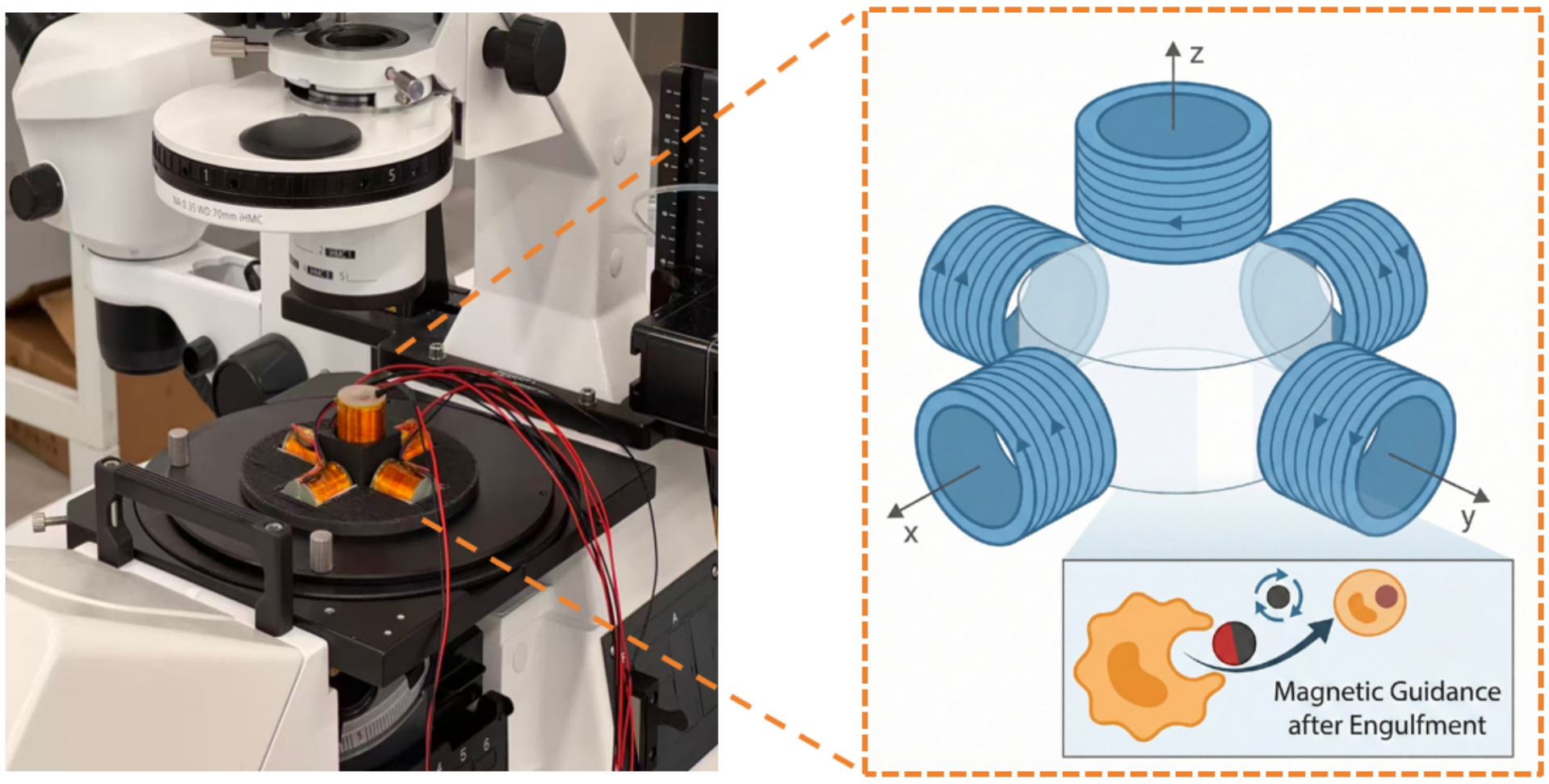

Fig. S5. Electromagnetic actuator setup used for *in vitro* actuation and magnetic navigation studies. Custom-built five-coil electromagnetic actuator system integrated with an inverted optical microscope for microroller and immunobot actuation. Inset showing a schematic illustration of each coil position and the sample space.

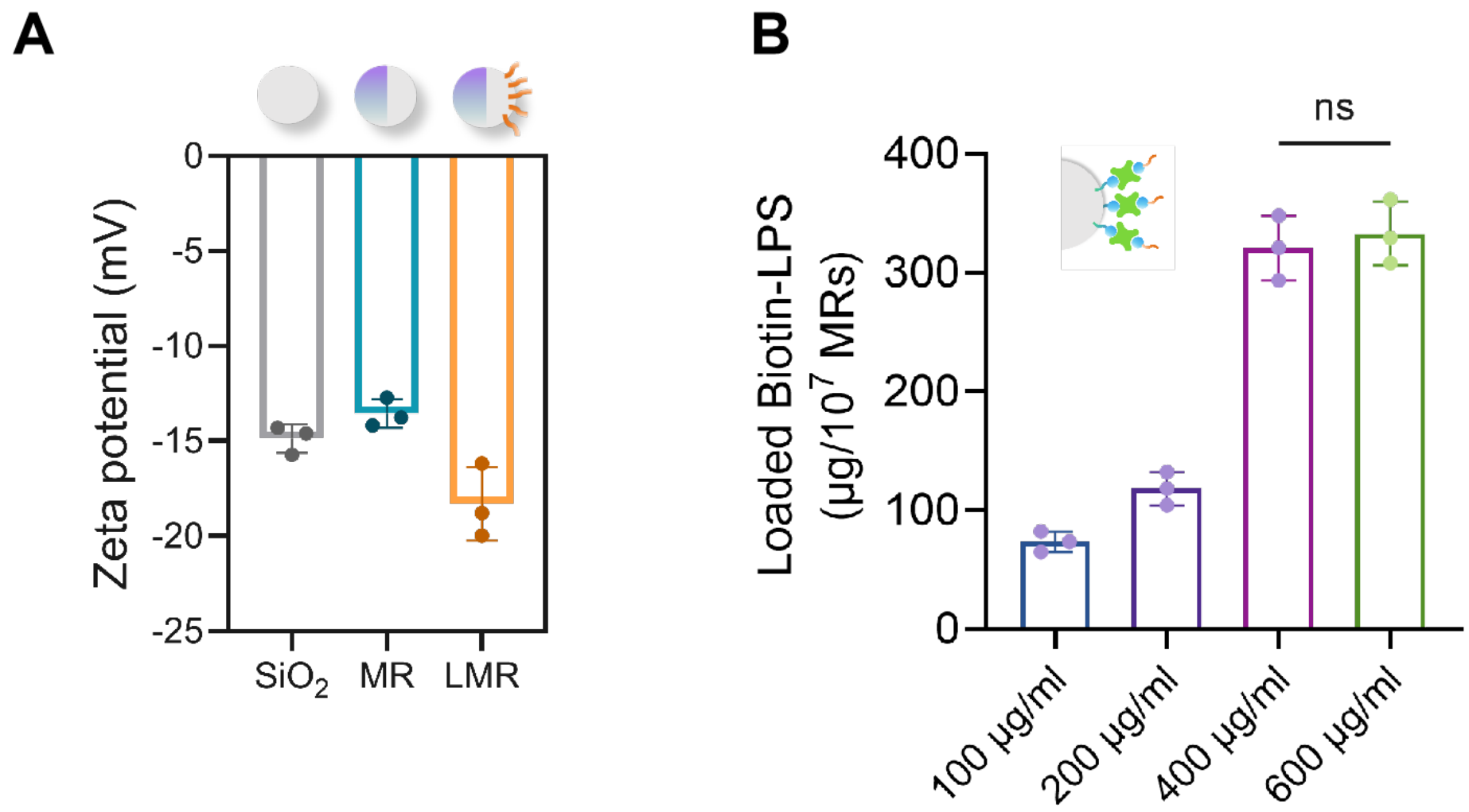

Fig. S6. Physicochemical validation of LPS-engineered Janus FePt microrollers. (A) Zeta potential alterations of the bare 5 μm SiO_2 microspheres, FePt-sputtered Janus microrollers (MR), and LPS-engineered microrollers (LMR) dispersed in PBS (pH 7.4). The distinct negative shift in the LMR group indicates the successful introduction of negatively charged LPS molecules onto the microroller surface. (B) Loading capacity of biotin-LPS on Janus microrollers dependent on initial LPS input. Data are presented as mean ± SD (n = 3).

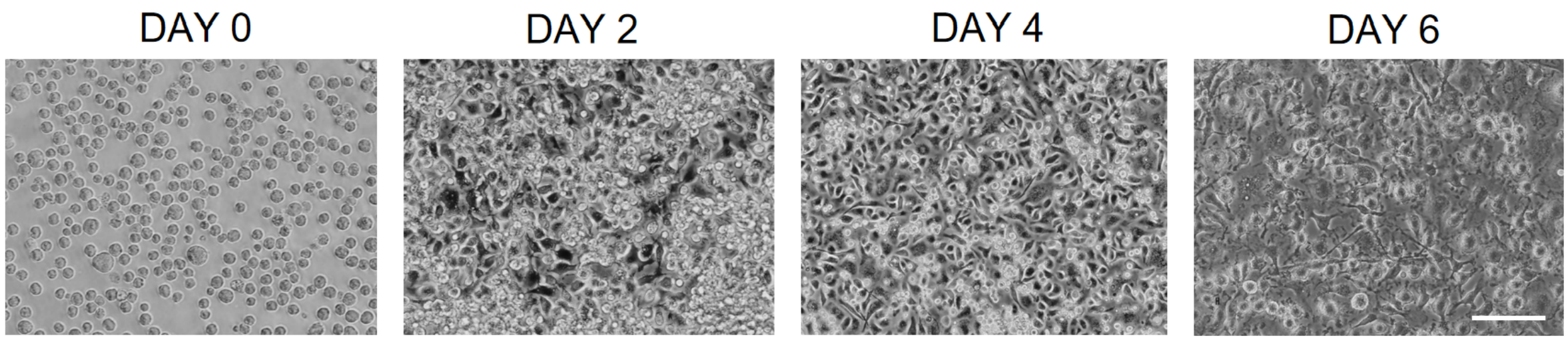

Fig. S7. Differentiation of bone marrow progenitor cells into macrophages. Morphological evolution of the cells at day 0, 2, 4, and 6 during the *in vitro* differentiation process. Scale bar, 50 µm.

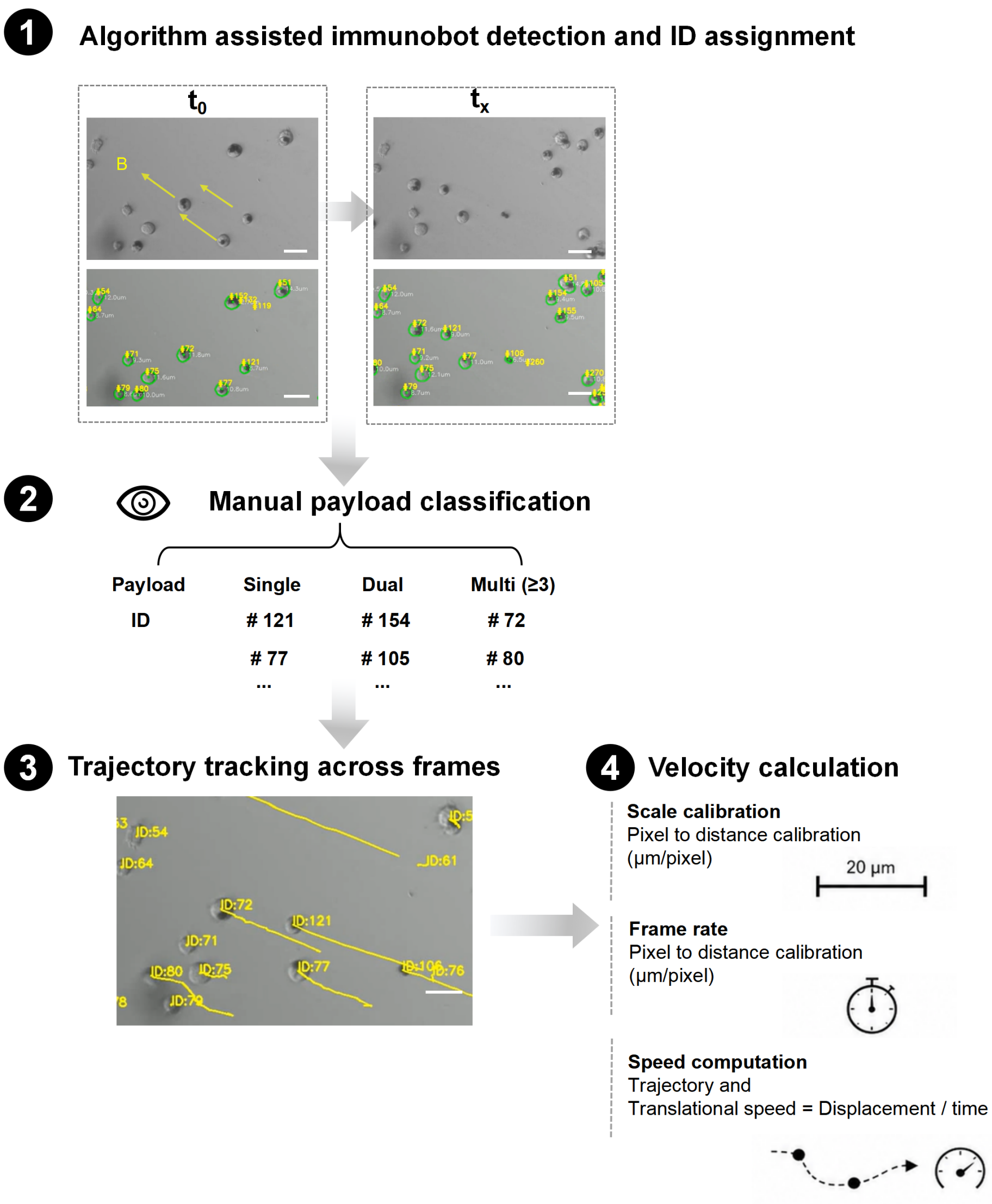

Fig. S8. Workflow for immunobot payload-resolved motion analysis. Tracking-assisted workflow for payload classification, trajectory tracking, and translational velocity calculation. Scale bar, 20 µm.

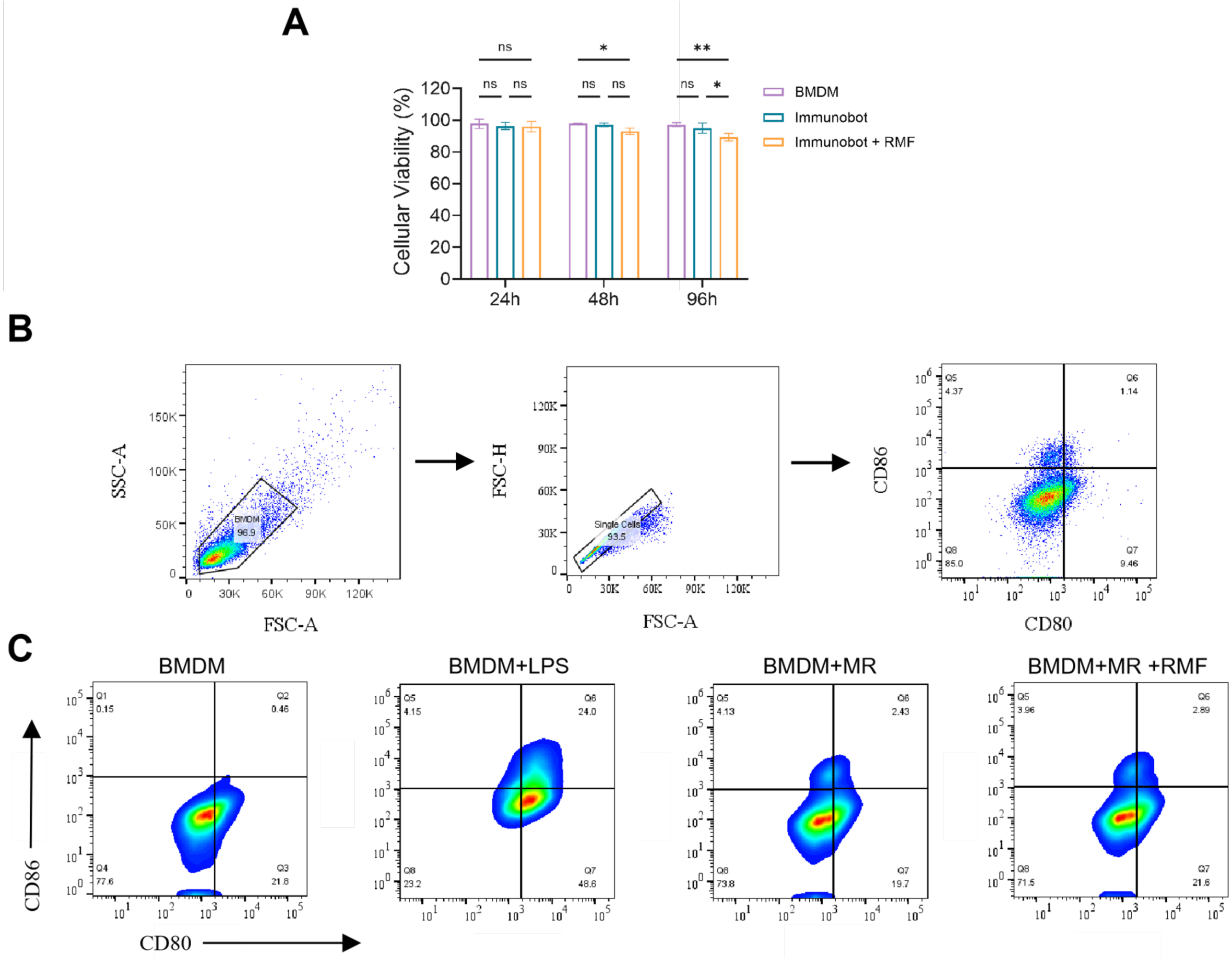

Fig. S9. Effect of external magnetic rotation and steering on cell viability and phenotype. (A) Viability results after rotating and steering at the step-out frequency (30 Hz) for 30 min. (B to C) FCM gating strategy and results indicating continuous rotation under RMF (30 Hz, 30 min) has a negligible effect on BMDM phenotype.

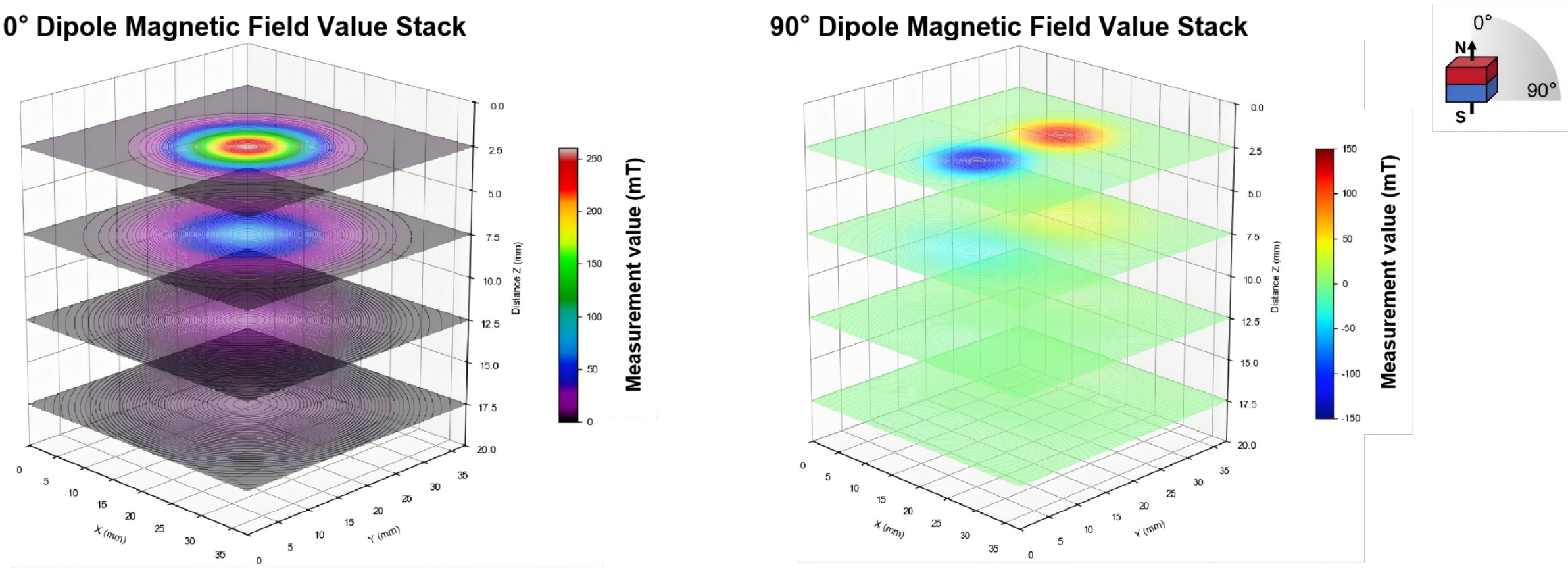

Fig. S10. Effective magnetic field strength of the permanent magnets as a function of distance. This magnet was used for the immunobots' flow-resistant retention and transwell penetration experiments. The figure shows the magnet at two angles over distance ($Z_{max} = 17.5$ mm).

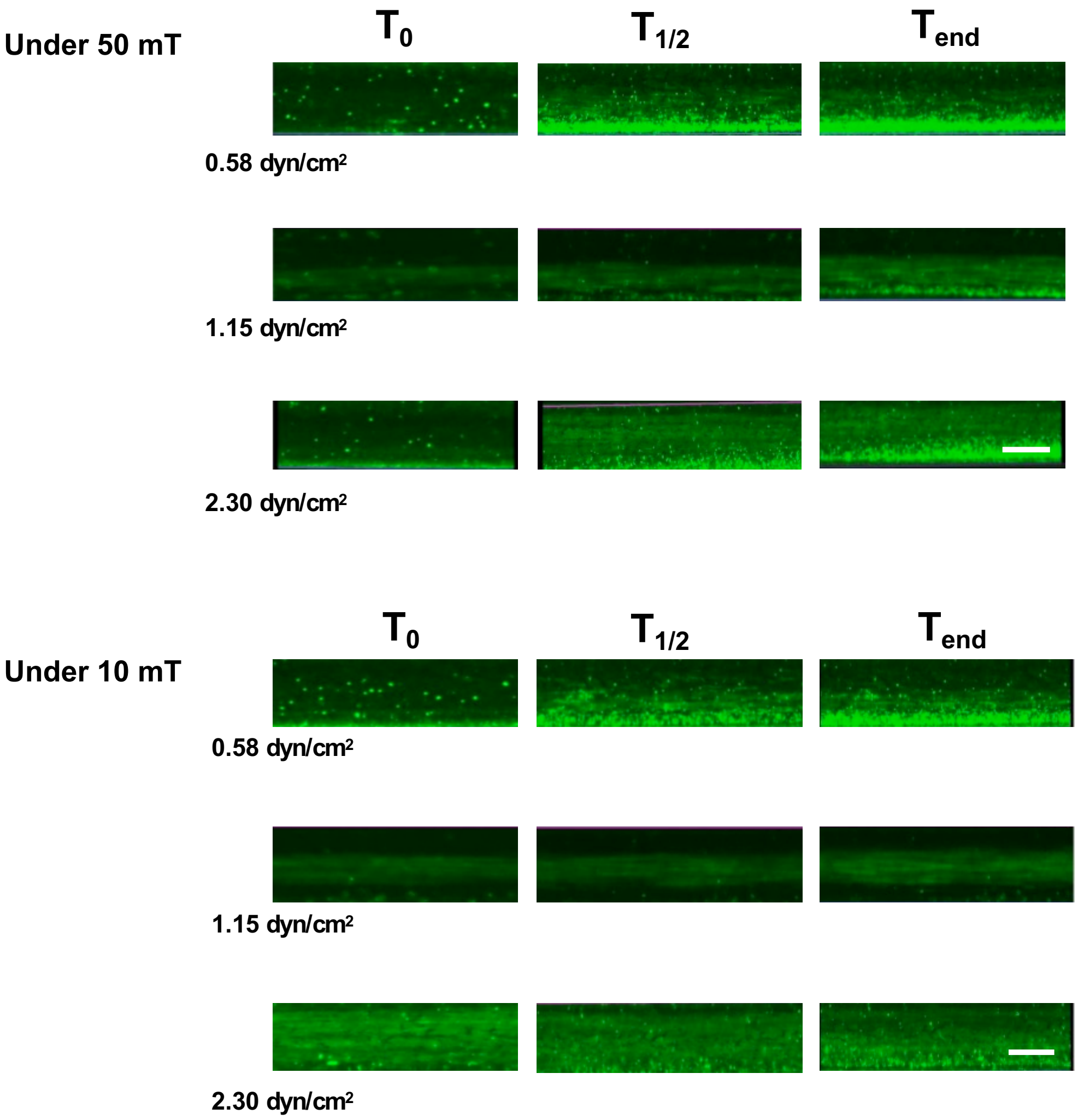

Fig. S11. Representative fluorescence time-lapse retention images of immunobots under the application of 50 and 10 mT magnetic field.

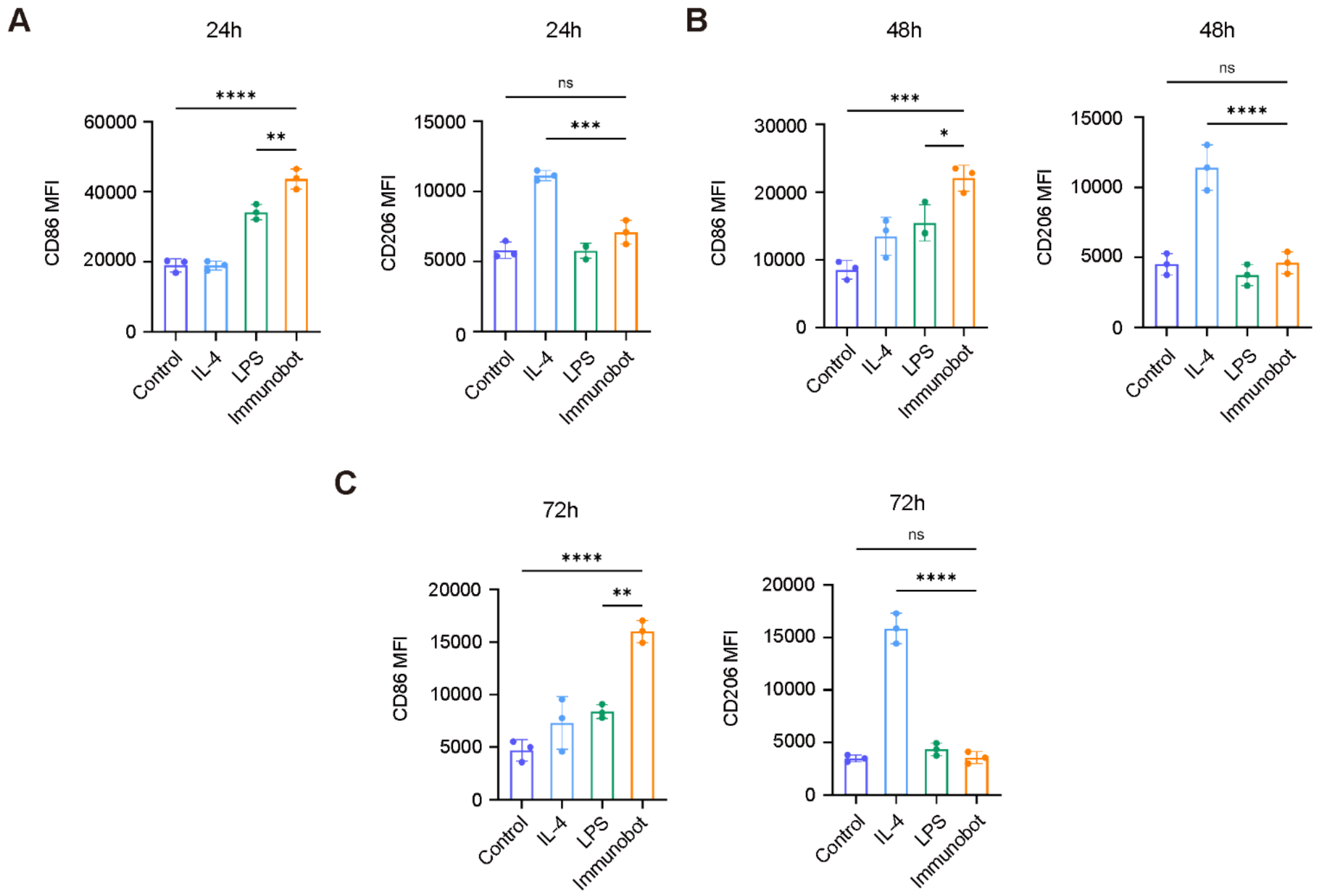

Fig. S12. Flow cytometric analysis of CD86 and CD206 expression in BMDMs at 24, 48, and 72 h. BMDMs were treated under the indicated conditions and analyzed by flow cytometry for CD86 and CD206 expression. Quantitative results show the MFI of CD86 and CD206 at 24 h (A), 48 h (B), and 72 h (C). Data are presented as mean ± SD, n = 3. Statistical analysis was performed using one-way ANOVA with Tukey's multiple comparisons test. *P < 0.05, **P < 0.01, ***P < 0.001, ****P < 0.0001; ns, not significant.

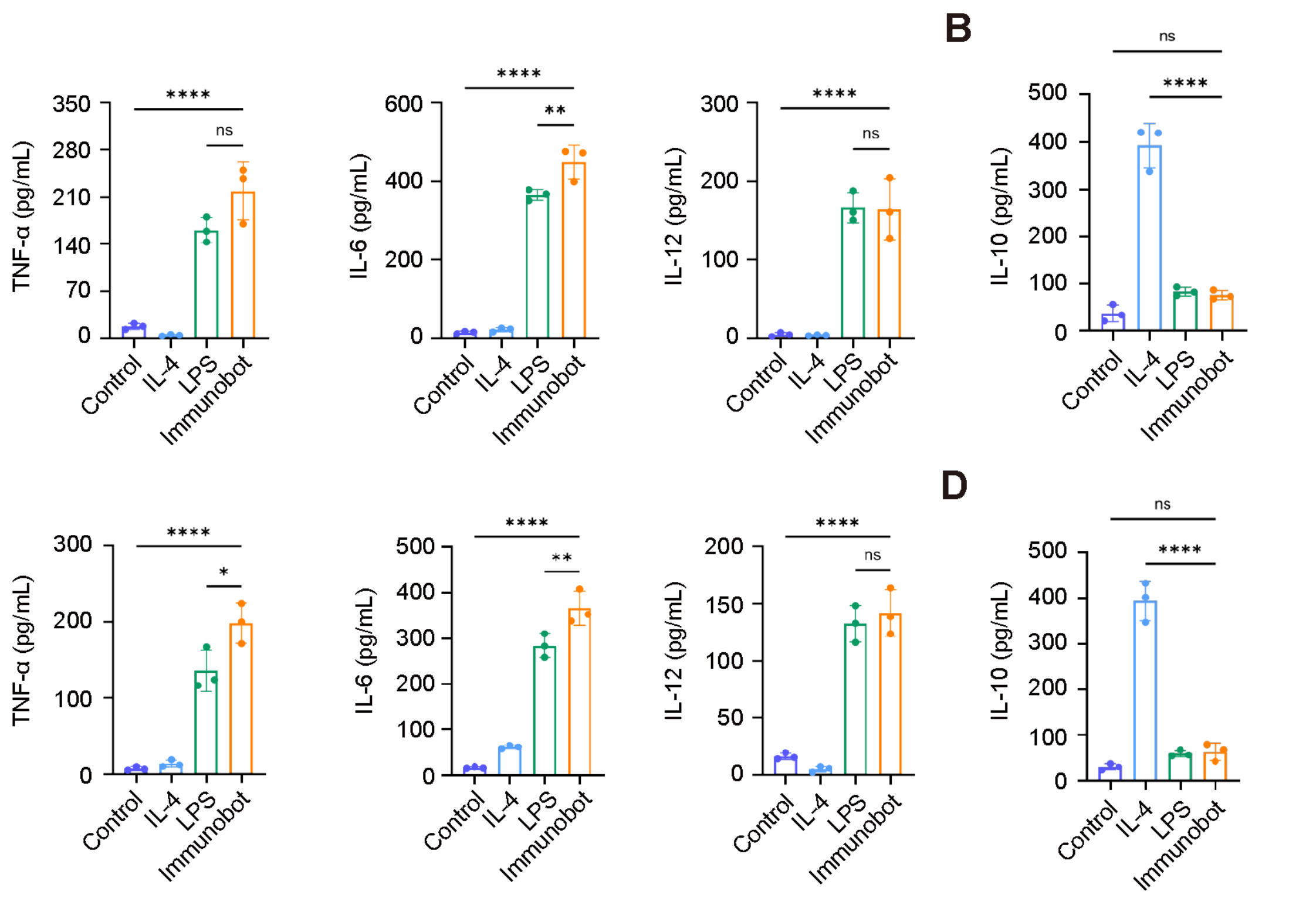

Fig. S13. ELISA analysis of cytokine secretion in BMDMs under the indicated conditions. (A) Secretion levels of M1-associated cytokines TNF-α, IL-6, and IL-12 at 48 h. (B) Secretion level of the M2-associated cytokine IL-10 at 48 h. (C) Secretion levels of TNF-α, IL-6, and IL-12 at 72 h. (D) Secretion level of IL-10 at 72 h. Data are presented as mean ± SD (n = 3). Statistical analysis was performed using one-way ANOVA with Tukey's multiple comparisons test. **P* < 0.05, ***P* < 0.01, *****P* < 0.0001; ns, not significant.

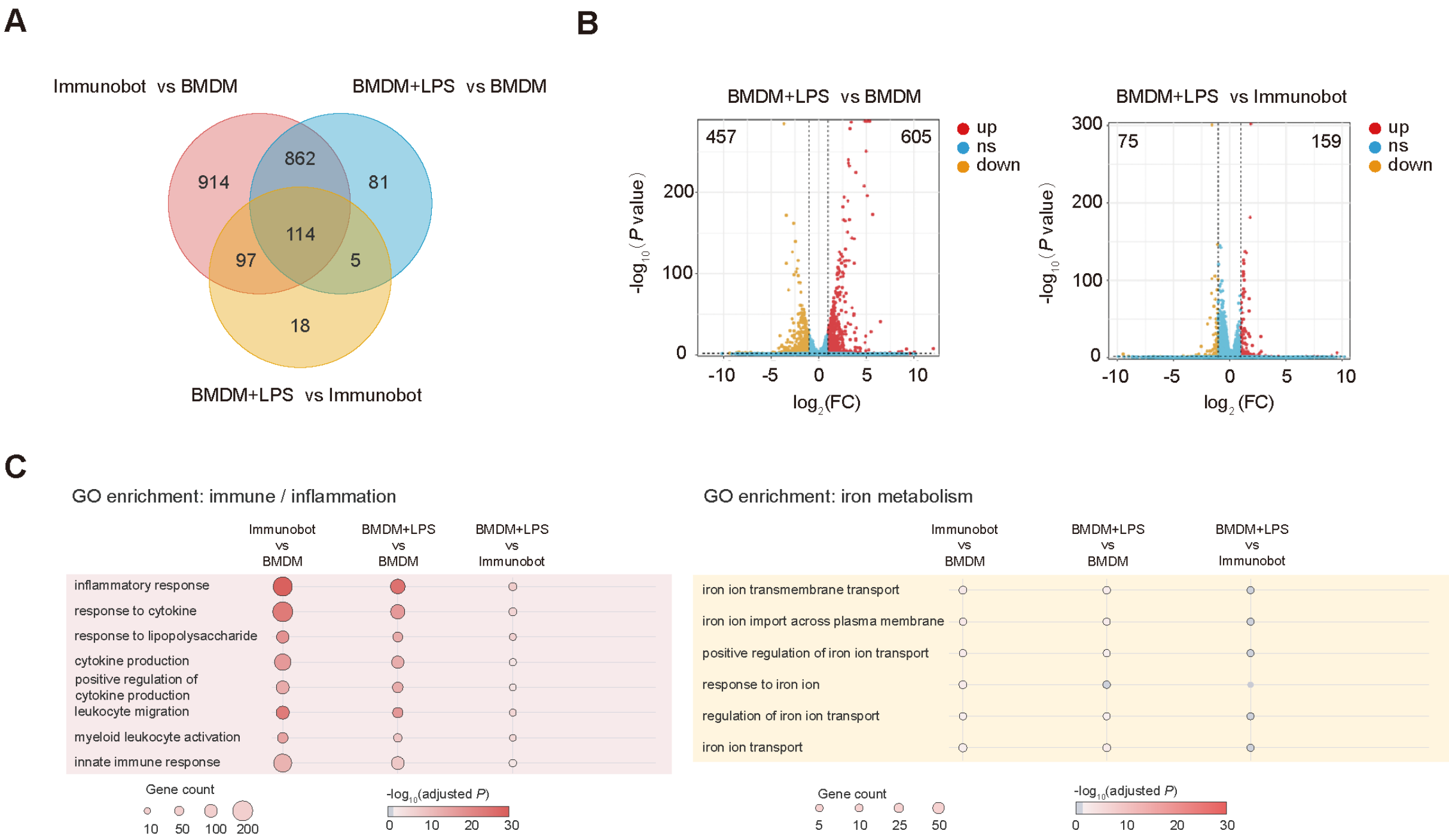

Fig. S14. RNA-seq profiling of immunobots. (A) Venn diagram showing shared and unique DEGs among the indicated comparisons. (B) Volcano plots showing DEGs in BMDM + LPS versus BMDM and BMDM + LPS versus immunobot. Red, orange, and blue dots indicate upregulated, downregulated, and non-significant genes, respectively. (C) GO enrichment analysis of immune/inflammatory and iron metabolism-related terms across the indicated comparisons. Bubble size indicates gene count, and color indicates $-\log_{10}$ adjusted *P* value.

Text S1. Sustained NF-κB activation induced by LMRs

We investigated the contribution of surface-bound LPS-mediated stimulation and FePt-derived iron signaling to sustained NF-κB activation induced by LMRs. To distinguish the inflammatory initiation mediated by LPS from the effects associated with FePt-derived iron signaling, we constructed LPS-SiO_2 as a non-iron LPS-bearing control material. The amount of biotin-LPS conjugation was adjusted to achieve comparable LPS loading between LPS-SiO_2 and LMRs, thereby allowing evaluation of the contribution of the FePt component to NF-κB signaling persistence under equivalent LPS stimulation conditions. The results showed that both LPS-SiO_2 and LMRs induced substantial NF-κB activation at early time points (fig. S15 F, G). Specifically, LPS-SiO_2 induced marked p65 phosphorylation at 2 h, indicating that surface-conjugated LPS effectively initiates NF-κB inflammatory signaling. However, with prolonged stimulation, NF-κB activation induced by LPS-SiO_2 gradually declined, whereas LMR-treated macrophages maintained elevated p65 phosphorylation levels, which persisted up to 12 h,

suggesting that LMRs possess a more sustained capacity to regulate inflammatory signaling. In contrast, MR treatment did not induce obvious NF-κB activation at most time points, with only a slight increase observed at 12 h. Together with the degradation and iron release characteristics of FePt under acidic phagolysosomal conditions (fig. S16), these results suggest that LMR-induced NF-κB activation follows a temporally regulated two-stage mechanism: the early phase is primarily initiated by surface-bound LPS-mediated inflammatory stimulation, whereas following particle internalization and FePt degradation, Fe^{2+}-associated redox signaling further enhances and sustains NF-κB activity. Therefore, LMRs do not induce prolonged NF-κB activation by acting as a persistent LPS reservoir; rather, they regulate macrophage inflammatory programming through the coordinated actions of early LPS-mediated inflammatory initiation and subsequent FePt-derived iron signaling-mediated amplification.

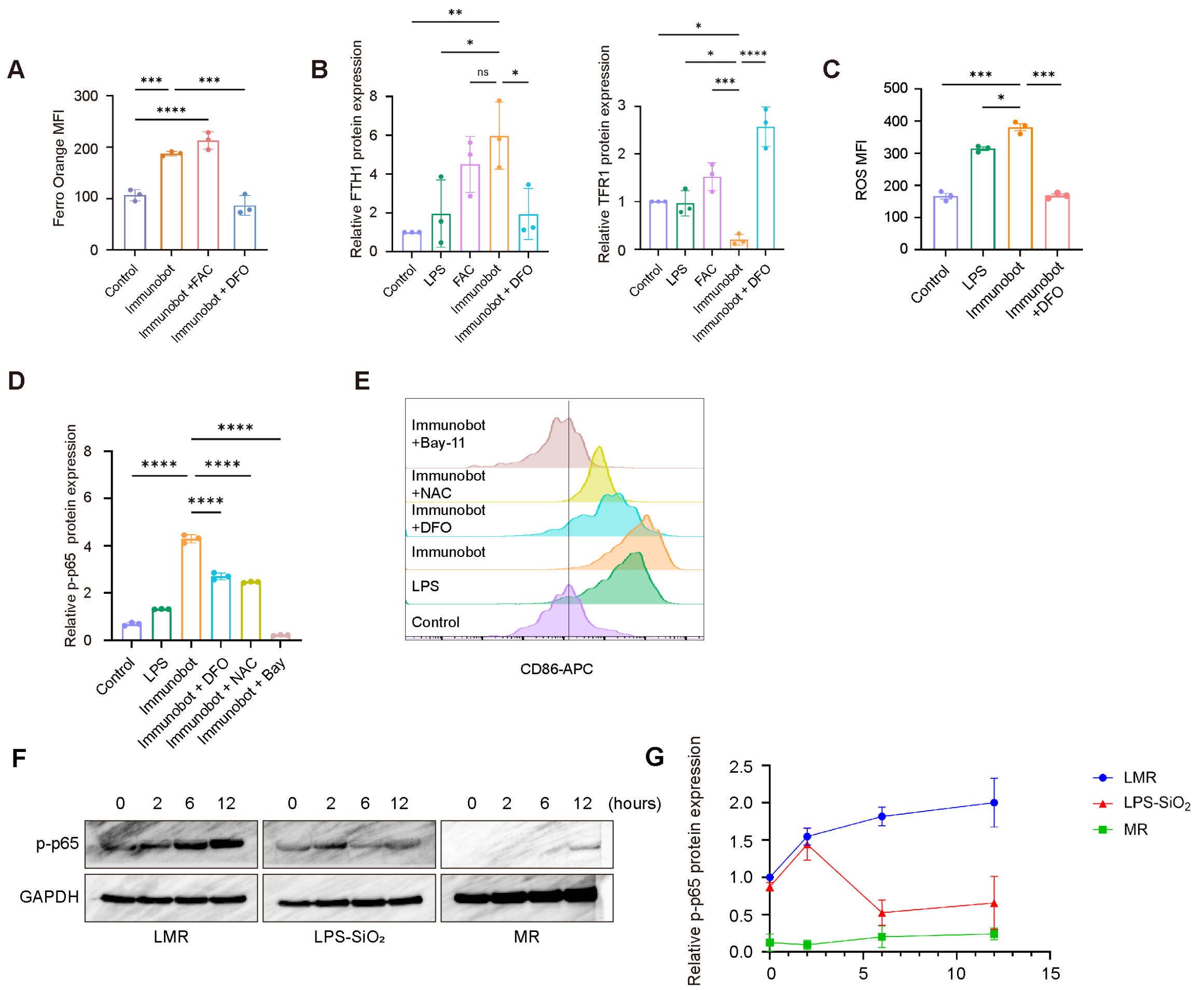

Fig. S15. Iron-associated activation of immunobots. (A) Quantification of intracellular labile iron levels by FerroOrange fluorescence intensity in the indicated groups. (B) Densitometric quantification of FTH1 and TFR1 Western blot bands normalized to GAPDH and expressed relative to the control group. (C) Quantification of intracellular ROS levels by fluorescence intensity in the indicated groups. (D) Densitometric quantification of phosphorylated p65

Western blot bands normalized to GAPDH and expressed relative to the control group. (E) Representative flow cytometry histograms showing CD86 expression after treatment. (F) Representative Western blot analysis of phosphorylated NF-κB p65 (p-p65) in macrophages following treatment with LMR, LPS-SiO_2, or MR for 0, 2, 6, and 12 h. GAPDH served as the loading control. (G) Densitometric quantification of p-p65 expression relative to GAPDH. Data are presented as mean ± SD, with dots representing independent biological replicates, n = 3. Statistical significance was determined by one-way ANOVA with Tukey's multiple comparisons test. *$P < 0.05$, **$P < 0.01$, ***$P < 0.001$, and ****$P < 0.0001$; ns, not significant.

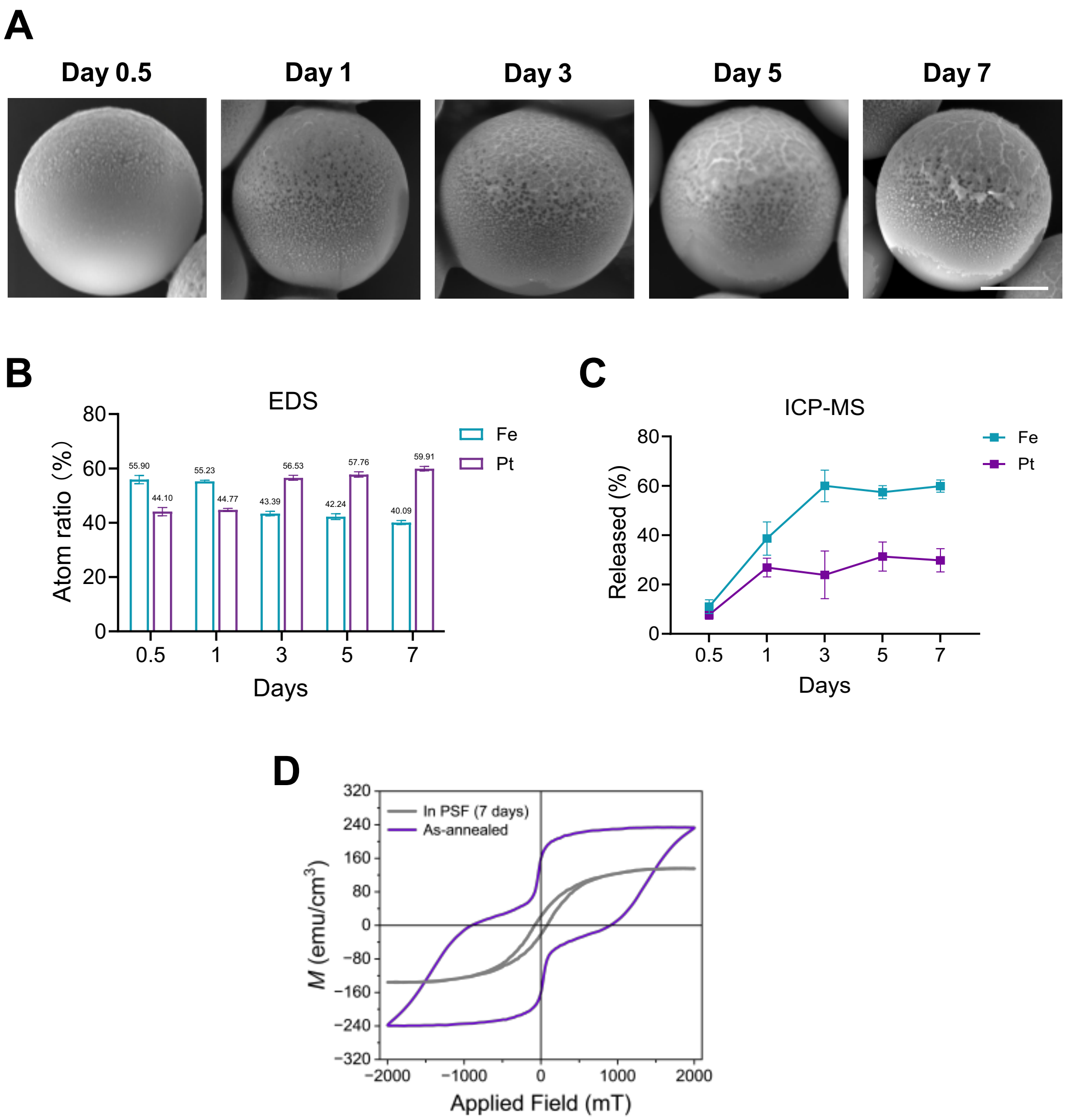

Fig. S16. Analysis of acid-triggered surface corrosion and preferential Fe leaching from FePt microrollers in phagolysosome simulating fluid (PSF). FePt microrollers were incubated in acidic PSF (pH ≈ 4.5) at 37 °C for 0.5, 1, 3, 5, and 7 days under shaking. (A) SEM images showing progressive surface roughening and localized pitting/crack-like features on the L10-FePt coating during acid exposure. Scale bar, 2 µm. (B) EDS analysis of the residual

coating reveals a time-dependent decrease in Fe atomic percentage and a corresponding increase in Pt atomic percentage. (C) ICP-MS analysis showing the released fractions of Fe and Pt at each time point, defined as the elemental content in the supernatant divided by the total elemental content recovered from the supernatant and pellet. These results indicate partial acid etching of the FePt coating with preferential Fe dissolution and relative Pt enrichment of the remaining surface. (D) VSM hysteresis loop showing the magnetic properties change after 7 days incubation within PSF.

Text S2. Evaluation of iron transfer via extracellular vesicles

To further evaluate whether EV-associated pathways contribute to immunobot-mediated iron transfer to tumor cells, we performed an EV secretion inhibition experiment. Specifically, GW4869 (S7609, Selleck), an inhibitor commonly used to suppress EV release, was applied in the immunobot treatment system, and its effects on tumor-cell iron accumulation and ferroptosis-related phenotypes were examined. The results showed that immunobot treatment markedly increased the intracellular FerroOrange fluorescence intensity in tumor cells, indicating elevated labile Fe^{2+} accumulation. In contrast, GW4869 treatment significantly attenuated the immunobot-induced increase in intracellular Fe^{2+} levels (fig. S17 F-G). Furthermore, C11-BODIPY staining demonstrated that immunobot treatment substantially increased the oxidized/reduced C11-BODIPY ratio, whereas inhibition of EV release by GW4869 markedly reduced this lipid peroxidation response (fig. S17 H-I).

These results indicate that inhibition of EV-associated secretion processes reduces immunobot-induced iron accumulation and lipid peroxidation in tumor cells, supporting the involvement of EV-associated pathways in immunobot-mediated iron redistribution and ferroptotic induction.

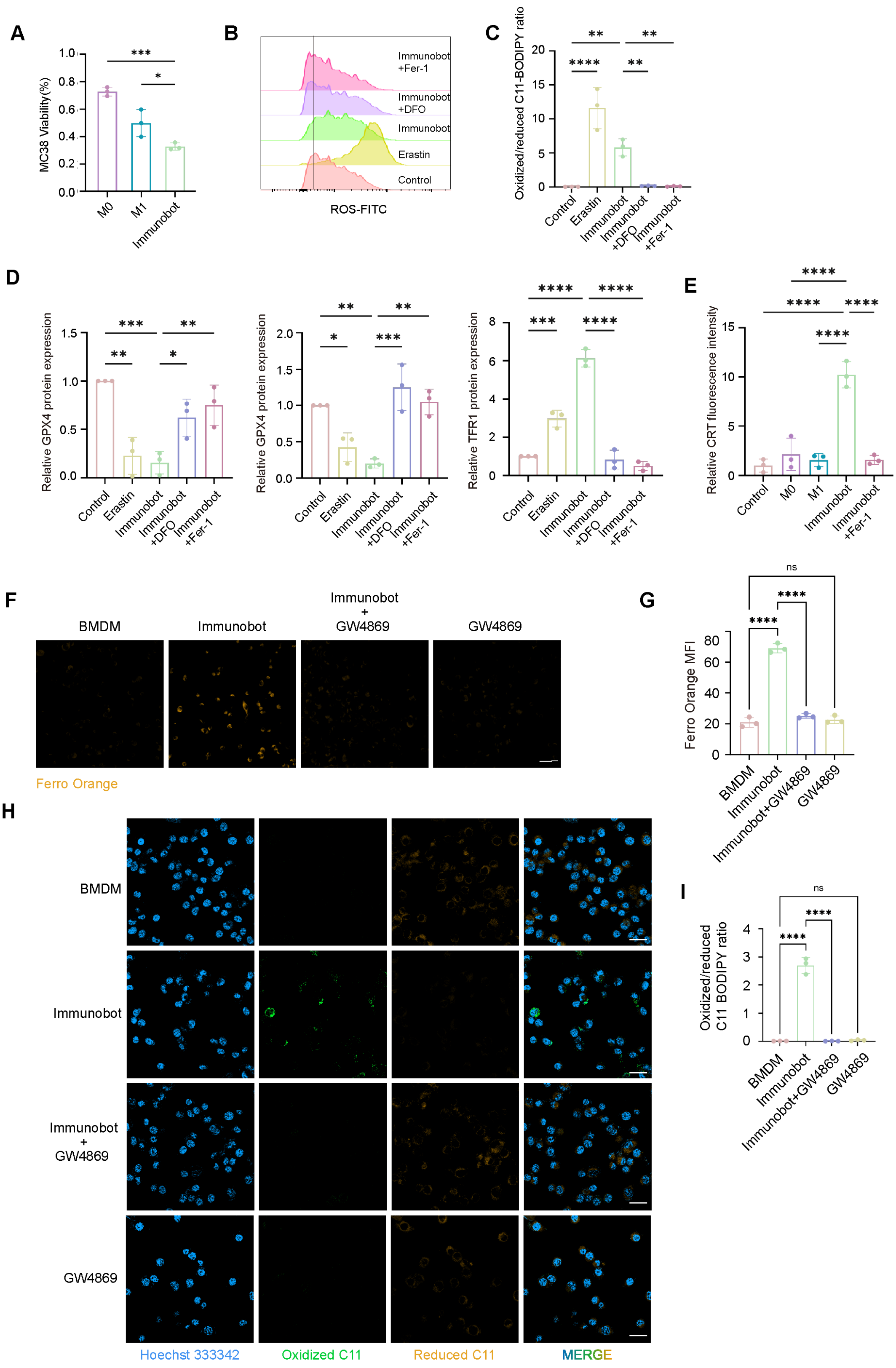

Fig. S17. Immunobots induce ferroptotic death and immunogenic signaling in MC38 tumor cells. (A) Viability of MC38 cells after treatment with M0 macrophages, M1 macrophages, or immunobots. (B) Representative flow cytometry histograms showing ROS-

FITC fluorescence in MC38 cells treated with control, erastin, immunobots, immunobots plus DFO, or immunobots plus Fer-1. (C) Quantification of lipid peroxidation by the oxidized/reduced C11-BODIPY ratio in MC38 cells under the indicated treatments. (D) Quantification of ferroptosis defense and iron-homeostasis proteins, including GPX4, TFR1, and FTH1, in MC38 cells after the indicated treatments. (E) Quantification of CRT fluorescence intensity in MC38 cells treated with control, M0 macrophages, M1 macrophages, immunobots, or immunobots plus Fer-1. (F) Representative fluorescence images showing intracellular labile Fe^{2+} levels in MC38 tumor cells after non-contact Transwell co-culture with the indicated groups. BMDMs, Immunobot-loaded BMDMs, or Immunobot-loaded BMDMs treated with GW4869 (5 μM) were seeded in the upper chamber, while MC38 cells were cultured in the lower chamber. MC38 cells treated with GW4869 (2.5 μM) alone were included as a control. Intracellular Fe^{2+} levels were detected using FerroOrange staining. Scale bar, 50 μm. (G) Quantification of FerroOrange fluorescence intensity (mean fluorescence intensity, MFI) in MC38 cells from the indicated groups. (H) Representative fluorescence images showing lipid peroxidation levels in MC38 cells after non-contact Transwell co-culture with the indicated groups. Oxidized C11-BODIPY and reduced C11-BODIPY signals were detected to evaluate lipid oxidation status. Nuclei were counterstained with Hoechst 33342. Scale bar, 20 μm. (I) Quantification of the oxidized/reduced C11-BODIPY fluorescence ratio in MC38 cells from the indicated groups. Data are presented as mean ± SD, n = 3 biologically independent samples. Statistical significance was determined by one-way ANOVA with Tukey's multiple-comparisons test. *$P < 0.05$, **$P < 0.01$, ***$P < 0.001$, and ****$P < 0.0001$.

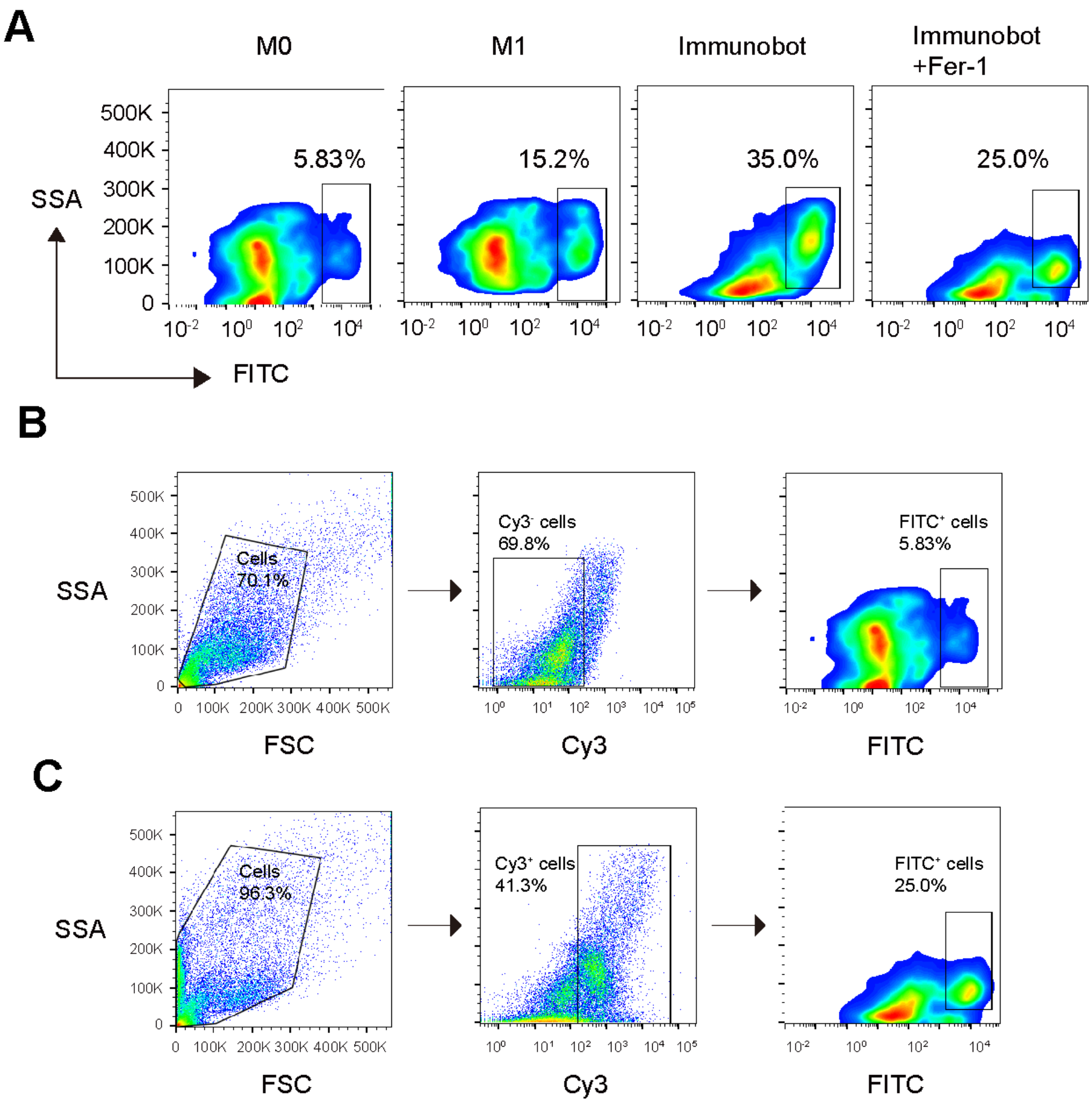

Fig. S18. Flow cytometric analysis of tumor cell phagocytosis. (A) Representative flow cytometry plots showing the phagocytosis of tumor cells by M0 macrophages, M1 macrophages, immunobots, and immunobots treated with Fer-1. The percentage of FITC-positive cells represents the phagocytic population. (B, C) Representative gating strategies for M0/M1 macrophages and immunobots, respectively. Cells were gated based on FSC/SSC, followed by Cy3-positive cell selection and FITC-positive cell quantification.

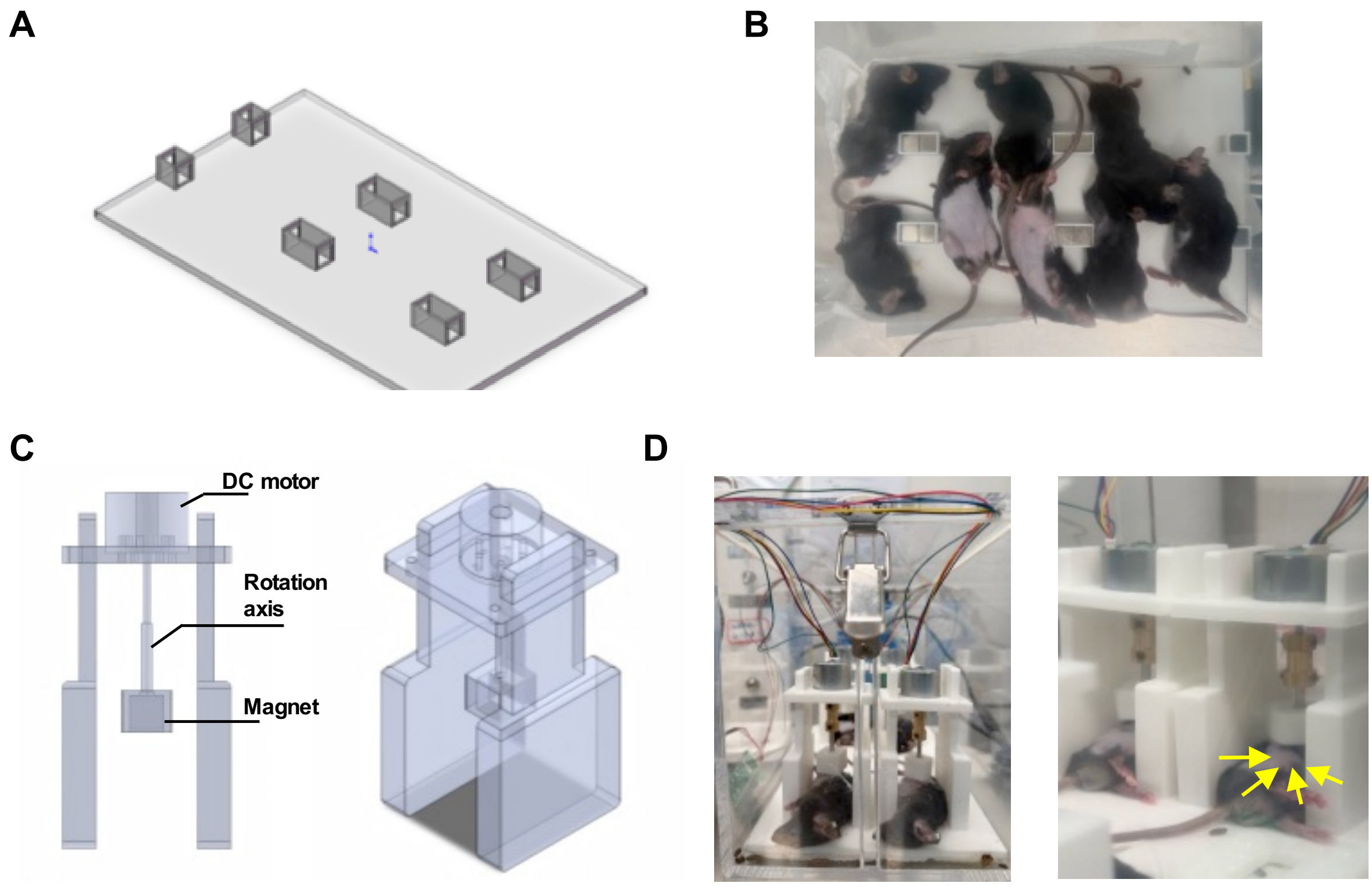

Fig. S19. ***In vivo*** **magnetic actuation setups.** (A) Permanent magnet fixation model used to apply gradient magnetic field to multiple mice at a time. (B) Representative operation image showing eight mice receiving gradient magnetic field. (C) Computer-aided design of rotating magnetic field application setup. DC motor connected to a permanent magnet. (D) Photograph of RMF application to multiple mice at a time to enhance immunobots penetration.

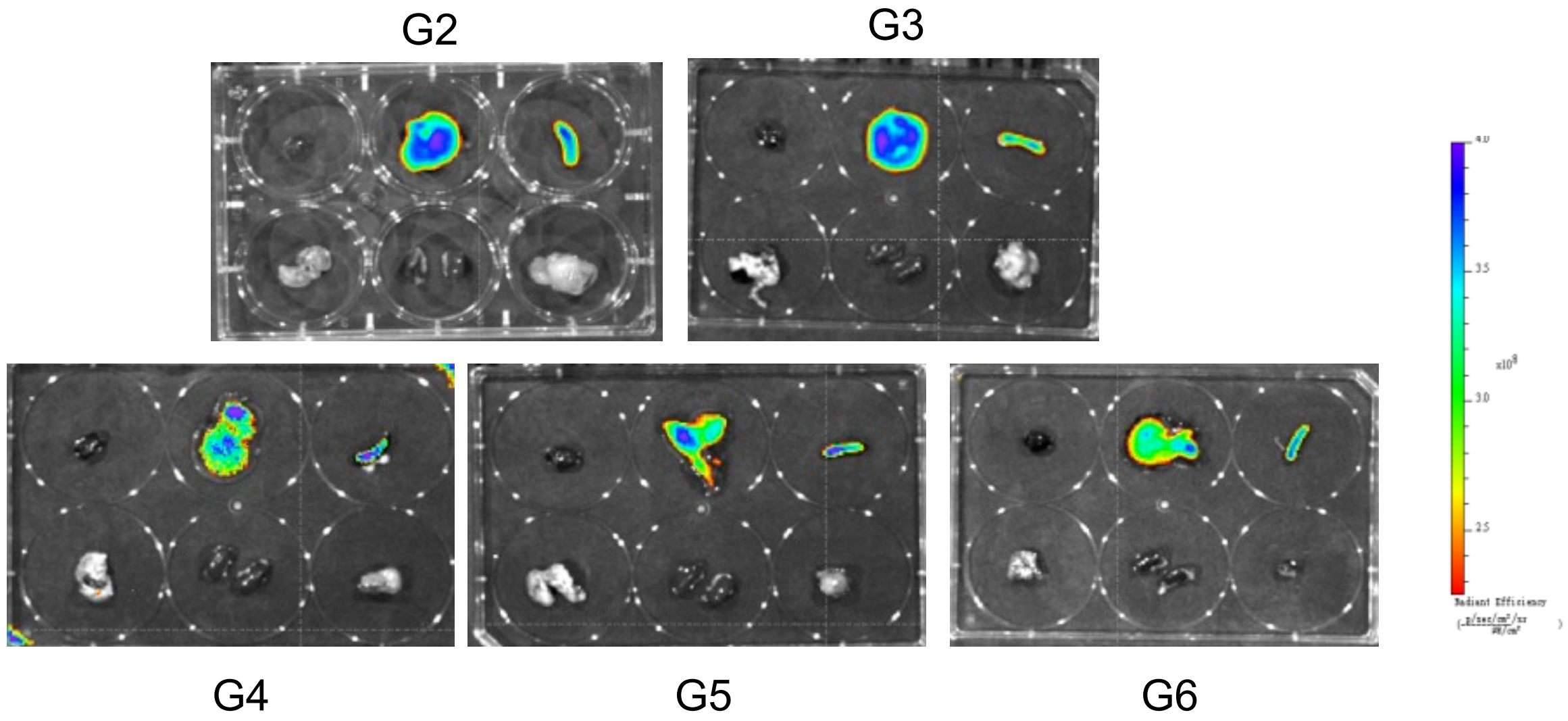

Fig. S20. ***Ex vivo*** **biodistribution of DiD-labeled formulations in major organs and tumors at the study endpoint**. In each six-well plate, the top row contains the heart, liver, and spleen, and the bottom row contains the lung, kidney, and tumor.

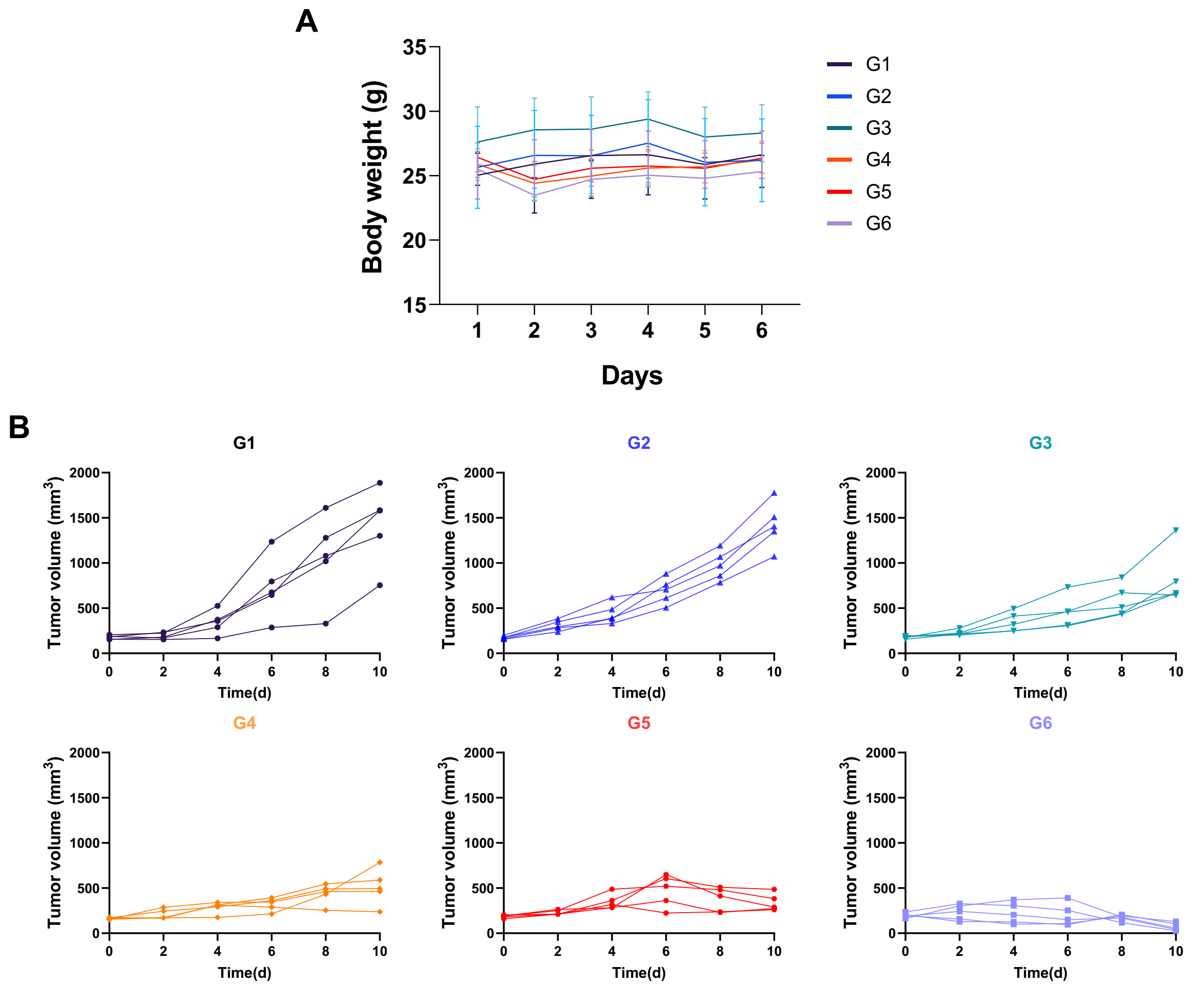

Fig. S21. Assessment of body weight and normalized tumor growth during treatment. (A) Changes in mouse body weight across the indicated treatment groups. (B) Tumor growth curves normalized to the initial tumor volume on day 0 across all six groups.

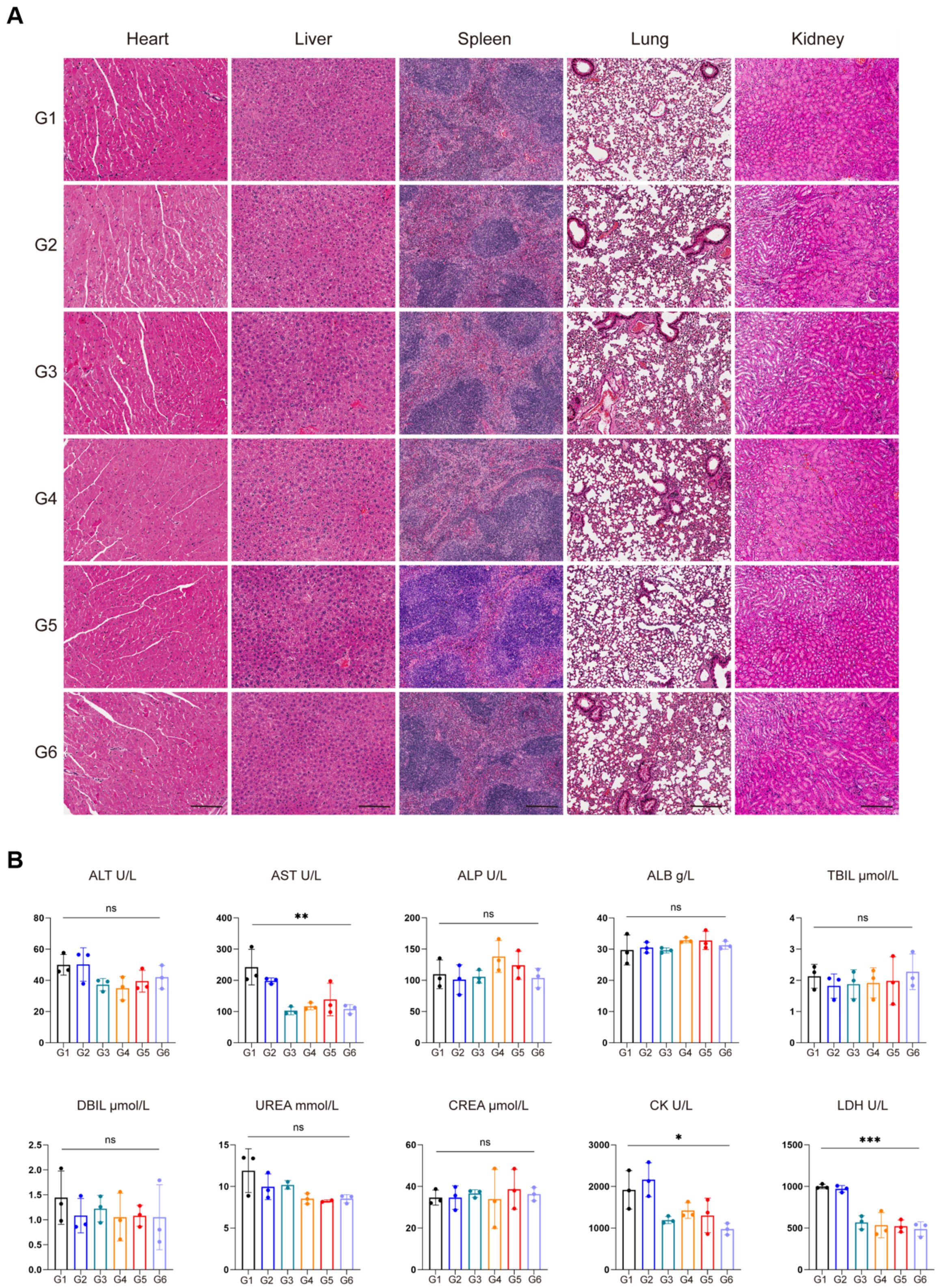

Fig. S22. *In vivo* biocompatibility and biosafety evaluation. (A) Representative H&E-stained sections of major organs (heart, liver, spleen, lung, and kidney) from different treatment groups. Scale bar, 100 μm. (B) Serum biochemical parameters at the sample collection time point in different treatment groups, including ALT, AST, ALP, ALB, TBIL, DBIL, UREA, CREA, CK, and LDH.

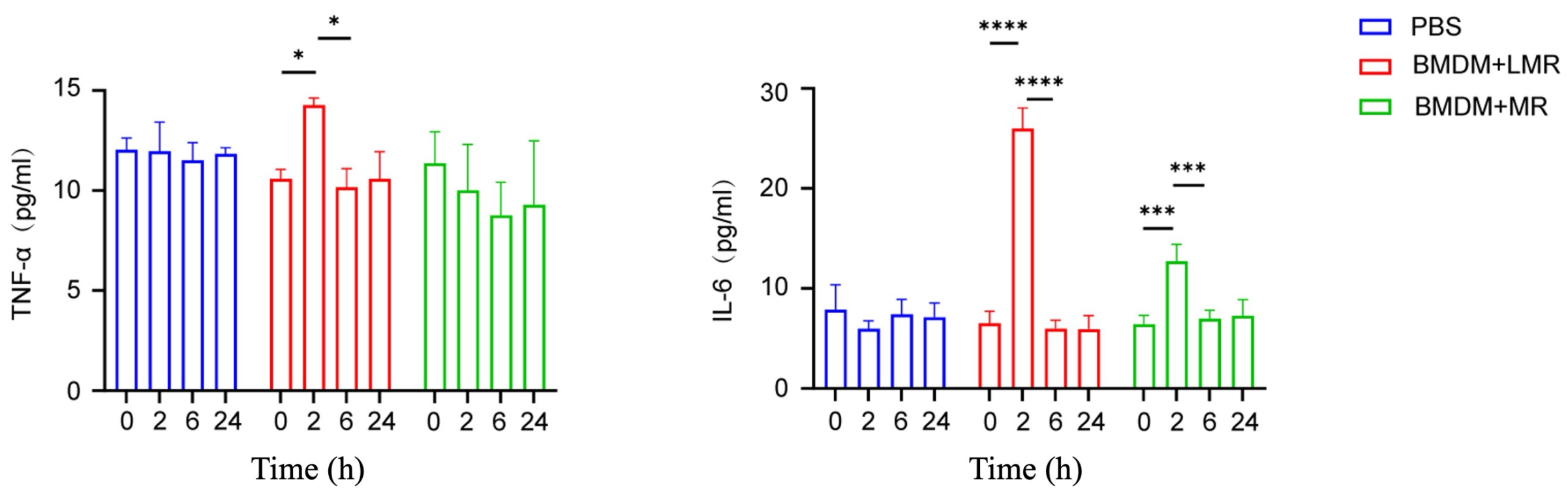

Fig. S23. Early systemic inflammatory responses following intravenous administration of immunobots. Serum levels of TNF-α and IL-6 were measured by ELISA at 0, 2, 6, and 24 h after intravenous administration of PBS, BMDM+LMR (immunobot), or BMDM+MR. Data are presented as mean ± SD. Statistical significance was determined by two-way ANOVA followed by a multiple-comparisons test. *P < 0.05, ***P < 0.001, and ****P < 0.0001.

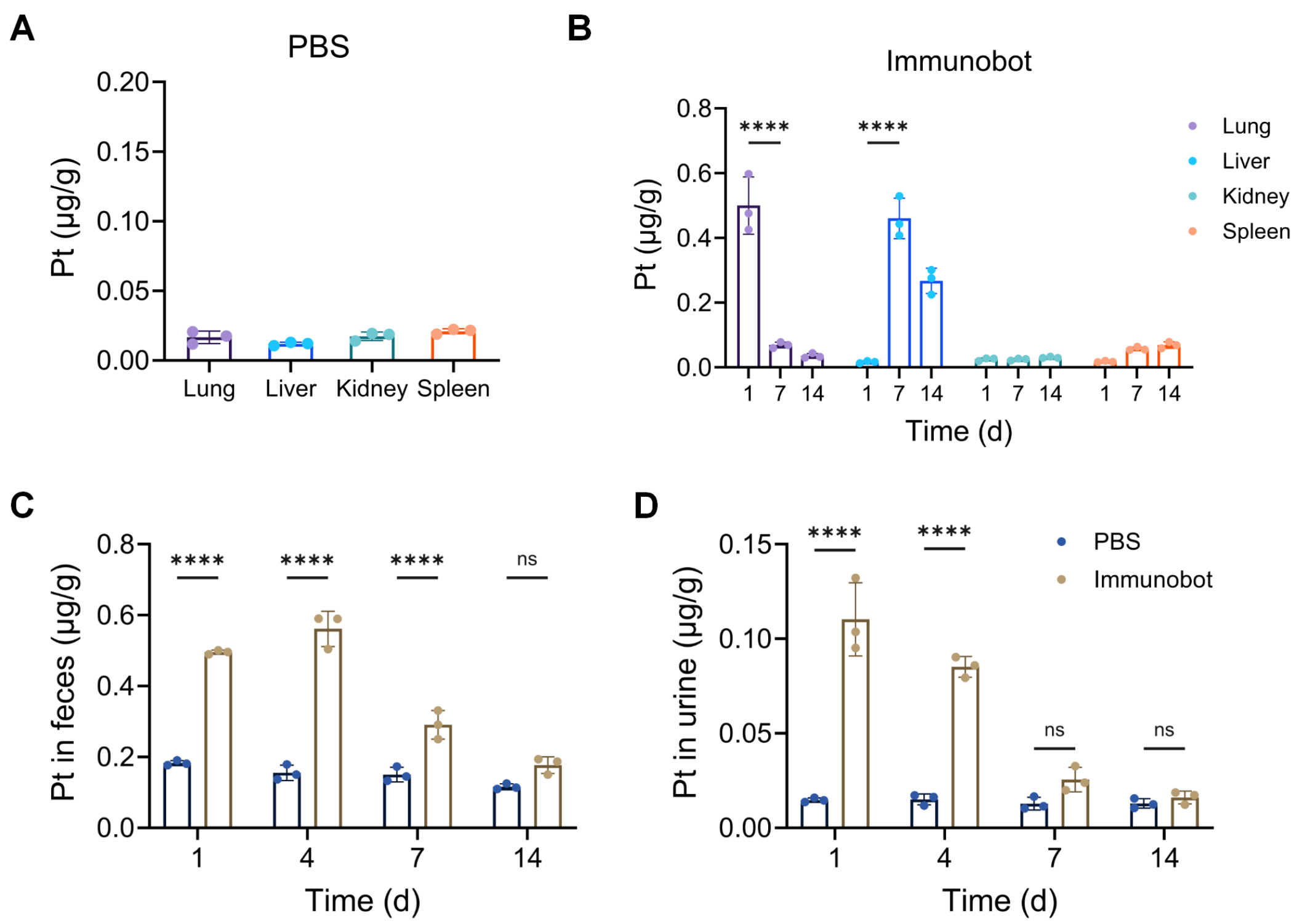

Fig. S24. Time-dependent biodistribution and excretion profiles of immunobot-associated Pt. (A) Baseline Pt concentrations in the corresponding organs of PBS-treated control mice. (B) Pt concentrations in the lung, liver, kidney, and spleen on days 1, 7, and 14 after intravenous administration of immunobots. Pt concentrations in feces (C) and urine (D) collected from PBS-

and immunobot-treated mice during separate 24-h sampling windows on days 1, 4, 7, and 10 after administration. Data are presented as mean ± SD. *P < 0.05, ***P < 0.001, and ****P < 0.0001.

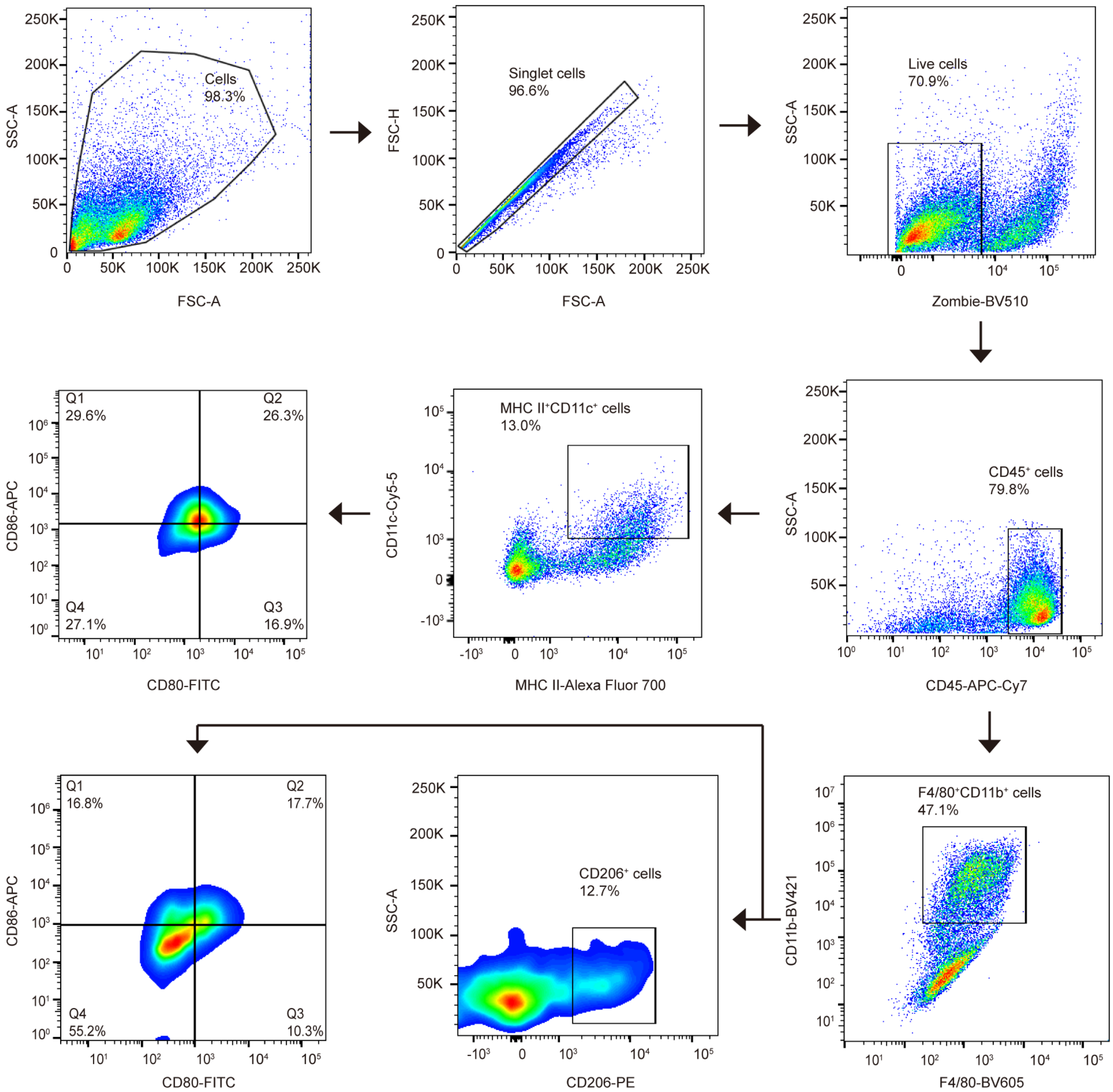

Fig. S25. Flow cytometry gating strategy for tumor-infiltrating macrophages and dendritic cells (DCs). Live $CD45^+$ immune cells were gated to identify MHC II^+CD11c^+ DCs and $F4/80^+CD11b^+$ macrophages, followed by analysis of CD80/CD86 and CD206 expression.

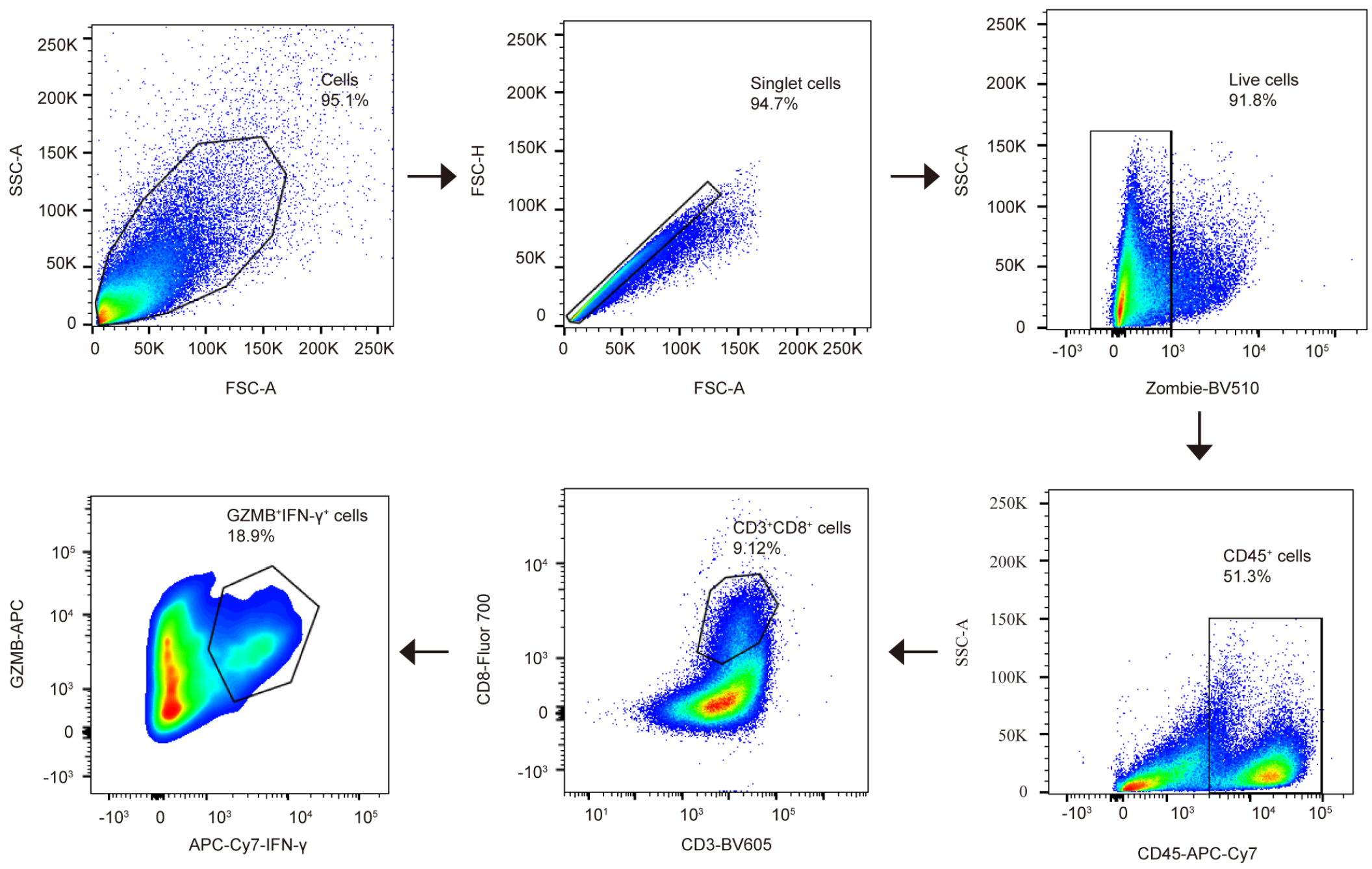

Fig. S26. Flow cytometry gating strategy for tumor-infiltrating CD8$^+$ T cells. Live CD45$^+$ immune cells were gated to identify CD3$^+$CD8$^+$ T cells, followed by analysis of granzyme B (GZMB) and IFN-γ expression.

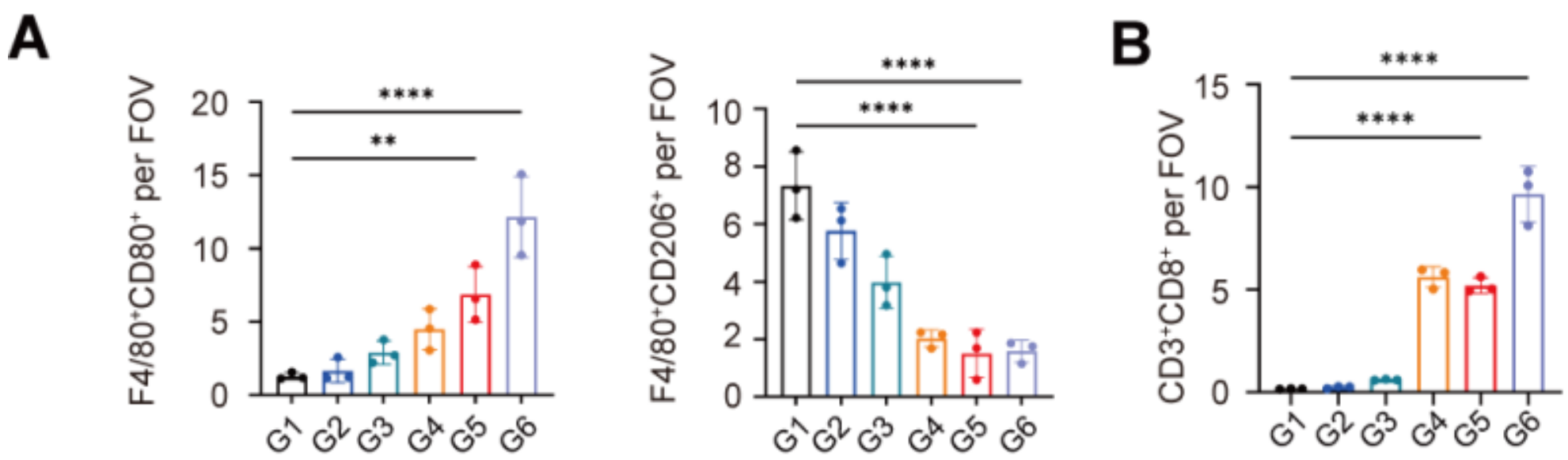

Fig. S27. Immunofluorescence quantification of macrophage polarization and CD8$^+$ T-cell infiltration. (A) Quantification of F4/80$^+$CD80$^+$ and F4/80$^+$CD206$^+$ cells per field of view (FOV) across groups G1 to G6. (B) Quantification of CD3$^+$CD8$^+$ cells per FOV across groups G1 to G6. Data are presented as mean ± SD; each dot represents one biological replicate. $^{**}P < 0.01$, $^{****}P < 0.0001$.

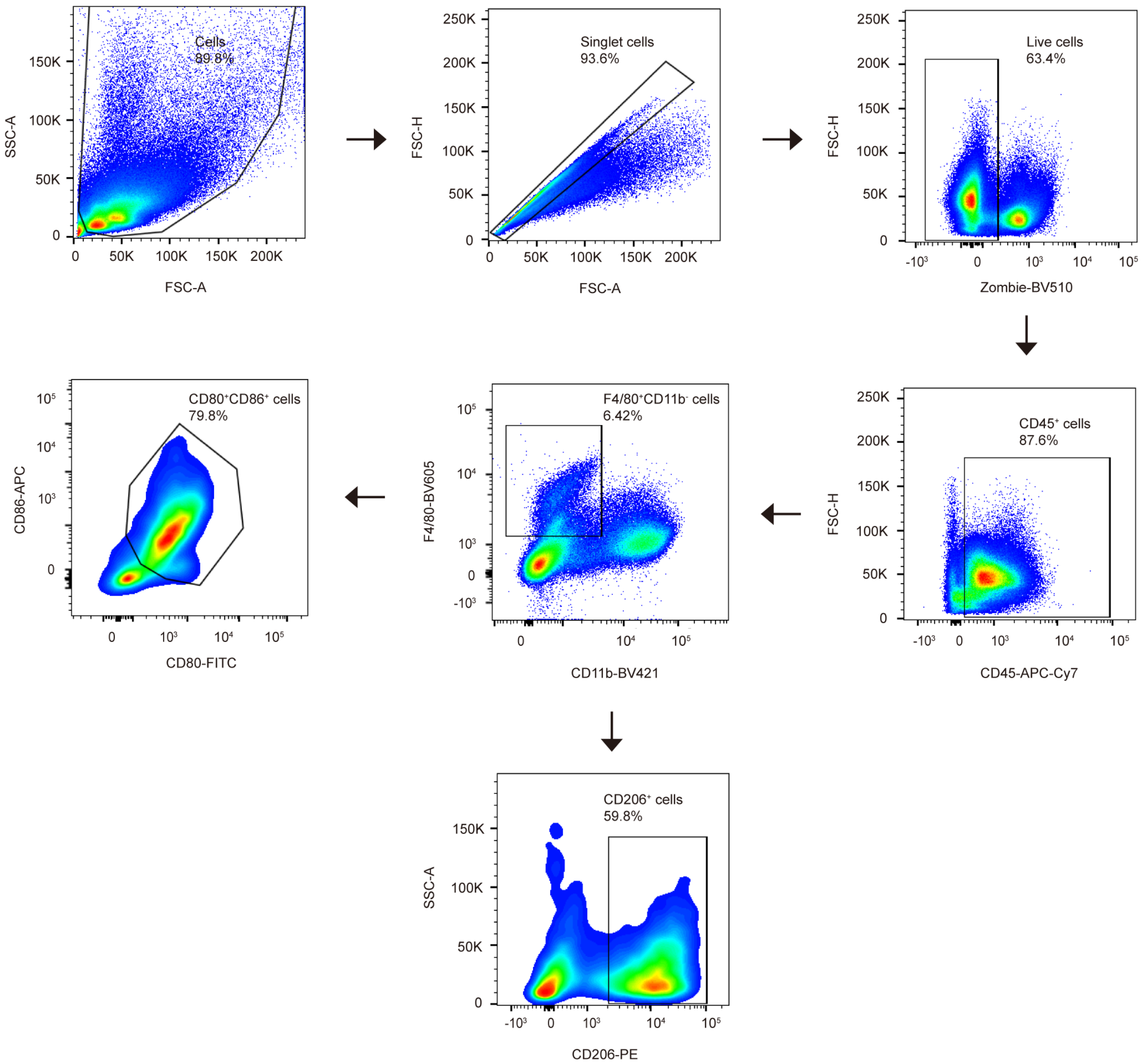

Fig. S28. Flow cytometric gating strategy for splenic macrophage phenotypes. Representative gating strategy for the analysis of splenic macrophage phenotypes. Cells were sequentially gated on the basis of FSC-A/SSC-A, singlets, live cells, and $CD45^+$ leukocytes. Splenic macrophages were defined as $CD45^+F4/80^+CD11b^-$ cells, followed by quantification of $CD80^+CD86^+$ M1-like macrophages and $CD206^+$ M2-like macrophages within this population. Percentages indicate representative frequencies within the corresponding parent gates.

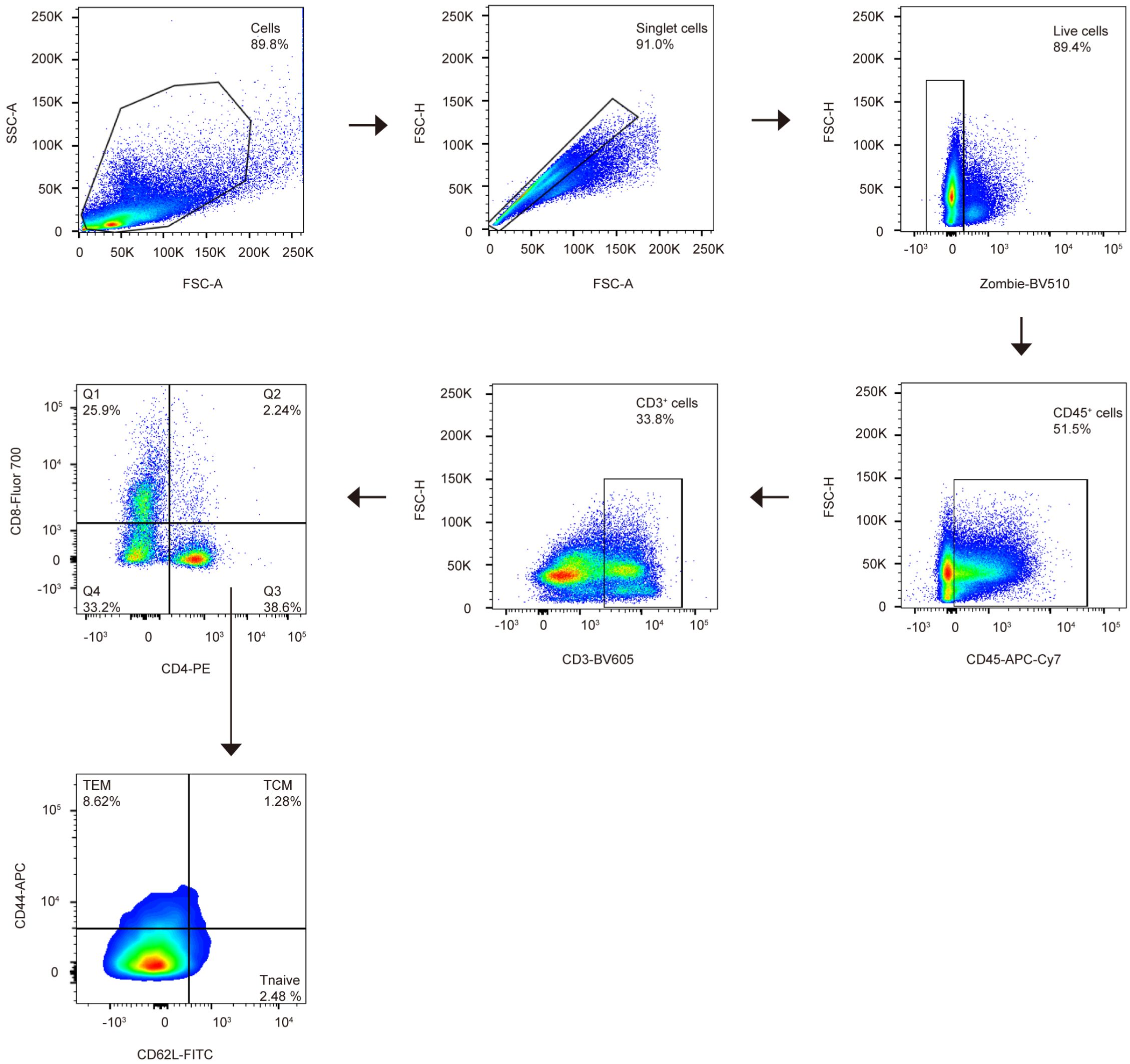

Fig. S29. Flow cytometry gating strategy for splenic T cells. Representative gating strategy used to identify splenic T-cell subsets. Cells were sequentially gated by FSC-A/SSC-A, singlets, live cells, and $CD45^+$ leukocytes. T cells were defined as $CD45^+CD3^+$ cells and further analyzed for $CD4^+$ subsets. Memory T-cell phenotypes were determined based on CD44 and CD62L expression, including effector memory T cells (T_EM, $CD44^+CD62L^-$), central memory T cells (T_CM, $CD44^+CD62L^+$), and naïve T cells (T_naive, $CD44^-CD62L^+$). Percentages indicate representative frequencies within the indicated parent gate.

Table S1. Sputtering power ratio between Fe/Pt targets and corresponding EDS atomic ratio. Data are mean ± SD， n=3. Values are presented as mean ± standard deviation from three different regions of each sample (n = 3). Atomic percentages were normalized to the sum of Fe and Pt (Fe + Pt = 100 at.%).

**Fe:Pt power ratio**	**Fe（at.%，mean ± SD）**	**Pt（at.%，mean ± SD）**
200:180	45.33 ± 2.18	54.67 ± 2.18
200:160	51.06 ± 1.35	48.94 ± 1.35
200:140	55.14 ± 1.42	44.86 ± 1.42
200:120	58.87 ± 2.27	41.13 ± 2.27

Table S2. Antibody information used in this study.

**Antigen**	**Company**	**Catalog number**	**Application**
Recombinant Anti-CD8 alpha antibody (Rabbit mAb)	Servicebio	GB15068-50	Immunofluorescence
Anti-F4/80 Rabbit pAb	Servicebio	GB113373-50	Immunofluorescence
Anti-Ki67 Rabbit pAb	Servicebio	GB111141-50	Immunofluorescence
Anti-Mouse Receptor/CD206 Rabbit pAb	Servicebio	GB113497-50	Immunofluorescence
Recombinant Anti-CD86 antibody (Rabbit mAb)	Servicebio	GB150054-50	Immunofluorescence
Anti-CD3 Rabbit pAb	Servicebio	GB11014-50	Immunofluorescence
Anti-CD31 Rabbit pAb	Servicebio	GB113151-50	Immunofluorescence
Recombinant Anti-4-Hydroxynonenal antibody (Mouse mAb)	Servicebio	GB150073-50	Immunofluorescence
Calreticulin Polyclonal antibody	Proteintech	27298-1-AF	Immunofluorescence
APC anti-mouse CD8a	BioLegend	100711	Flow cytometry
APC/Cyanine7 anti-mouse CD45	BioLegend	103115	Flow cytometry
Brilliant Violet 421™ anti-mouse/human CD11b	BioLegend	101235	Flow cytometry
PE anti-mouse CD4	BioLegend	100511	Flow cytometry
Brilliant Violet 605™ anti-mouse F4/80	BioLegend	123133	Flow cytometry
PerCP/Cyanine5.5 anti-mouse CD11c	BioLegend	117327	Flow cytometry
Brilliant Violet 605™ anti-mouse CD3 ε	BioLegend	100351	Flow cytometry
FITC anti-mouse CD80	BioLegend	104705	Flow cytometry
Alexa Fluor® 700 anti-mouse I-A/I-E	BioLegend	107621	Flow cytometry
APC-conjugated anti-mouse CD86	BioLegend	105011	Flow cytometry
PE-conjugated anti-mouse CD206	BioLegend	141705	Flow cytometry

APC anti-human/mouse Granzyme B Recombinant	BioLegend	372204	Flow cytometry
APC/Cyanine7 anti-mouse IFN-γ	BioLegend	505849	Flow cytometry
APC anti-mouse/human CD44	BioLegend	103011	Flow cytometry
FITC anti-mouse CD62L	BioLegend	104405	Flow cytometry
Zombie Violet™ Fixable Viability Kit	BioLegend	423113	Flow cytometry
TruStain FcX™ PLUS (anti-mouse CD16/32)	BioLegend	156603	Flow cytometry
Ferritin Heavy Chain Polyclonal Antibody	Proteintech	11682-1-AP	Western blot
CD71 Polyclonal Antibody	Proteintech	10084-2-AP	Western blot
GAPDH Monoclonal Antibody	Proteintech	60004-1-Ig	Western blot
Phospho-NF-kappaB p65 (Ser536) (93H1) Rabbit Monoclonal Antibody	Cell Signaling Technology	3033T	Western blot
GPX4 Monoclonal antibody	Proteintech	67763-1-Ig	Western blot

Supplementary Movies

Movie S1.

FePt Janus MR translation speed at different rotating frequency.

Movie S2.
Phagocytosis of LMRs by BMDMs.

Movie S3.

Comparison of the pre-magnetized and remagnetized immunobots' locomotion performance.

Movie S4.

Magnetic navigation of immunobots in diverse environments.

Movie S5.

Gradient field capture and flow-resistant retention of immunobots.

Movie S6.
In vivo magnetic field application system.

Movie S7.
Co-localized IVIS and micro-CT imaging of tumor targeting in mice.
